# Advances in quinoxaline derivatives: synthetic routes and antiviral efficacy against respiratory pathogens

**DOI:** 10.1039/d4ra04292a

**Published:** 2024-11-07

**Authors:** Fateme Dehnavi, Malihe Akhavan, Ahmadreza Bekhradnia

**Affiliations:** a Pharmaceutical Sciences Research Center, Department of Medicinal Chemistry, Mazandaran University of Medical Sciences Sari Iran abekhradnia@gmail.com abekhradnia@mazums.ac.ir

## Abstract

The study of quinoxalines as nitrogen-rich heterocyclic compounds has garnered substantial interest within scientific research owing to their multidimensional functionalization capabilities and significant biological activities. The scope of study encompasses their application as potent antiviral agents, particularly within the domain of respiratory pathologies—a topic of pivotal concern in this comprehensive review. They have several prominent pharmacological effects, such as potential influenza inhibitors, potential anti-SARS coronavirus inhibitors, potential anti-SARS-CO-2 coronavirus inhibitors, and miscellaneous respiratory antiviral activities. As a result, some of the literature has described many of these quinoxalines using various synthetic methods for their mentioned biological effects. In the present review, we provided insight into quinoxaline synthesis, structure–activity relationship (SAR), and antiviral activities, along with a compilation of recent studies. The article further encapsulates the gamut of past and ongoing research efforts in the design and synthetic exploration of antiviral scaffolds, with a pronounced emphasis on their strategic deployment against viral pandemics, contextualized against the tapestry of the recent COVID-19 outbreak. This illuminates the quintessential role of quinoxalines in the armamentarium against viral pathogens and provides a platform for the development of next-generation antiviral agents.

## Abstract 2

3D lung visualization showing COVID-19 infection areas alongside respiratory pathogens. The antiviral drug structure and comparison of healthy *vs.* infected lungs demonstrate how the drug could restore lung health.

## Graphical abstract

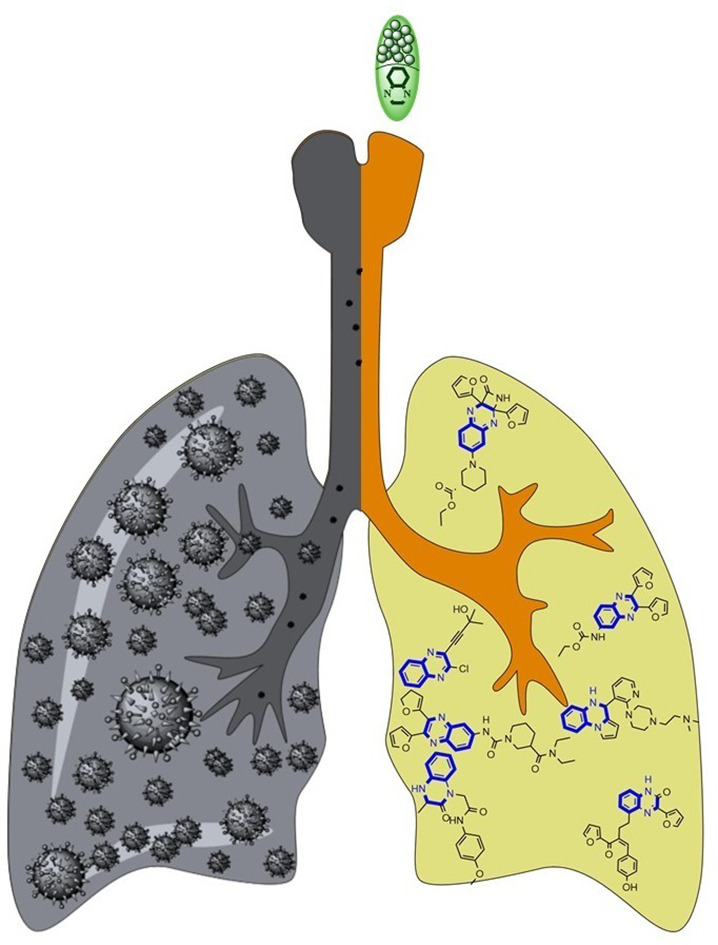

## 1. Introduction

The ceaseless pursuit of efficacious therapeutic agents persists as a formidable challenge within the medical field, not solely for pre-existing disorders but also in response to emergent crises, such as the recent emergence of COVID-19. During such exigencies, the protracted timespan required for the discovery, development, and regulatory approval of new pharmaceuticals intensifies the imperative for expeditious identification of viable candidates. This imperative has galvanized the exploration of innovative methodologies encompassing the repurposing of pre-existing pharmacophores and the *de novo* discovering of novel molecular scaffolds through scrutinizing currently approved medications. The antiviral design and synthetic capabilities manifested in select antiviral compounds have gained particular prominence against the backdrop of the latest SARS-CoV-2 pandemic and the spectre of future antiviral emergencies. We briefly address the existing literature and highlight the gap our review aims to fill, emphasizing the focused research on respiratory viruses and the inclusion of post-2020 studies.

Quinoxaline derivatives represent a pivotal class of heterocyclic compounds, distinguished by the substitution of nitrogen atoms for one or more carbons within the naphthalene core. Structurally, they comprise fused benzene and pyrazine rings, rendering a white crystalline solid with a melting point ranging from 29 to 30 °C and a molecular formula of C_8_H_6_N_2_. These compounds are typically purified chemically through distillation and exhibit solubility in water. As delineated in this discourse, various synthetic methodologies for quinoxaline derivatives encompass condensation reactions, cyclization processes, microwave-assisted synthesis, and other diverse strategies. The structural diversity of quinoxaline frameworks includes an array of derivatives, including but not limited to echinomycin analogs, carbonylamino-substituted quinoxalines, 2-chloroquinoxaline varieties, the antiviral agent Glecaprevir, as well as pyrazolo[1,5-*a*]quinoxaline, imidazo[1,5-*a*]quinoxaline, pyrrolo[1,2-*a*]quinoxaline, and pyridazino[4,5-*b*]quinoxalin derivatives.^1–6^

**Fig. 1:**
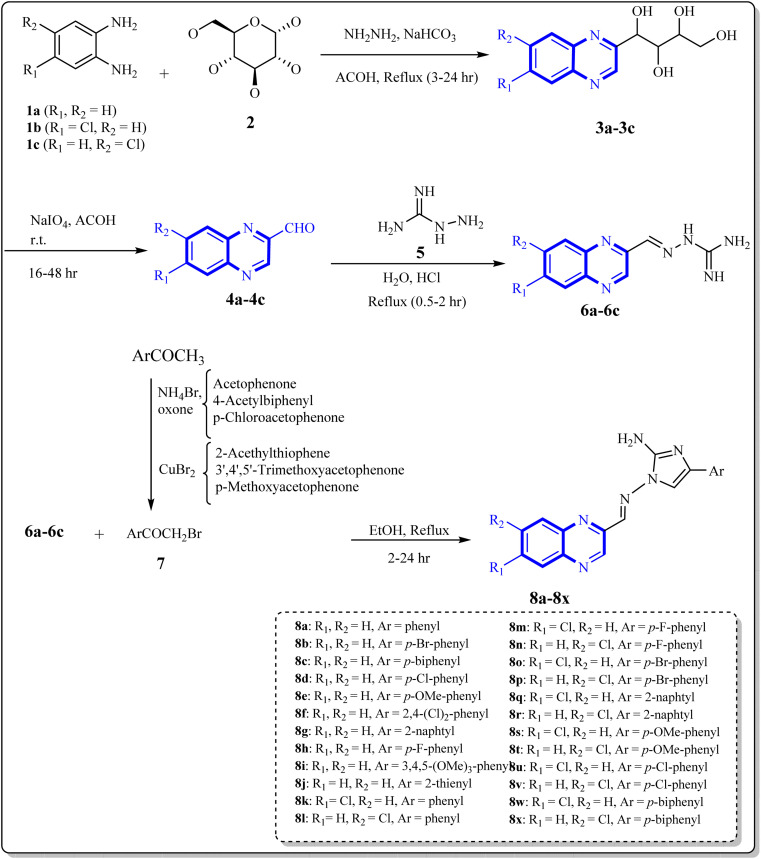
Synthesis of quinoxaline derivatives containing 2-aminoimidazoles.

**Fig. 2:**
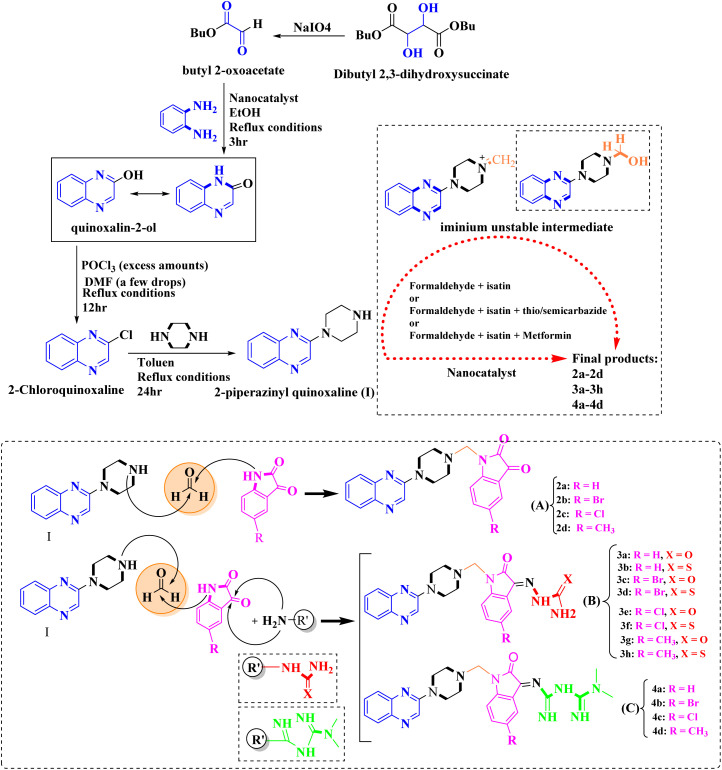
The final products are derived from 2-piperazinyl quinoxaline as the core scaffold, attached to the isatin-based Schiff bases of Metformin and/or thio/semicarbazones.

As one of the prominent useful motifs, the quinoxalines moiety featured in a huge number of chemosensors and synthetic pharmaceutical compounds by diverse properties, including fluoresence and anticancer properties as elucidated in our preceding investigation.^7–9^ In our previous work, a series of quinoxaline derivatives bearing 2-aminoimidazole were designed and synthesized (Fig. 1)^10^ These compounds were evaluated for their potential biological activities, and the results demonstrated significant effects at 50 μg mL^−1^ concentration with a noteworthy percentage of inhibition. Moreover in different research we reported some new methods, such as the green synthesis procedure and Mannich reactions, for the synthesis of various isatin-based Schiff bases and 2-piperazinyl quinoxaline core and 2-(piperazin1-yl) quinoxaline and other quinoxaline derivatives (Fig. 2).^11^ We reported one-pot multicomponent cyclo-condensation and Mannich reactions, shown in Fig. 2, with the aim of reducing the complications of using mineral or homogeneous organic catalysts and traditional methods.^10^ The reported methods were green synthesized, eco-friendly, non-toxic, economical, and easy-to-workup.^8,10^ Recently, our research team investigated an *in silico* study of quinoxaline motif against COVID-19.^11–13^ Following our recent field study, we are moving forward with a survey of quinoxaline and its activity on respiratory viruses like SARS-CoV-2.

In light of the recent pandemic, the current review pivots to an examination of the antiviral potential against respiratory pathogens. It posits quinoxaline derivatives as promising candidates for antiviral intervention, given their presence within the chemical structure of therapeutic agents such as echinomycin derivatives, Glecaprevir, and other molecules. The expansive scope of this review contemplates the utility of quinoxaline derivatives as prospective inhibitors of influenza viruses, anti-SARS and SARS-CoV-2 coronaviruses, alongside a range of other respiratory viral infections, underscoring their significance in the search for novel antiviral agents. The relentless march of infectious diseases, compounded by the emergence of the COVID-19 pandemic, has underscored the critical need for a rapid response in the development of antiviral agents. The protracted drug discovery process, from initial concept to regulatory approval, often impedes the ability to address immediate public health crises. In such scenarios, the scientific community is driven to identify alternative strategies that can shorten the pathway to effective treatments. One such strategy is re-investigating existing drugs and molecular scaffolds for potential antiviral properties—an approach that can significantly reduce development time and costs. Within this realm, heterocyclic compounds such as quinoxaline derivatives have garnered attention due to their broad pharmacological potential and the ease with which they can be modified to enhance their activity and specificity. These nitrogen-containing bicyclic compounds exhibit a range of biological activities, and their utility has already been affirmed in various therapeutic contexts, including cancer treatment. Exploring quinoxaline derivatives in the context of antiviral therapy is a logical extension, considering the urgency to develop agents that can contend with viral infections, particularly those of the respiratory tract. The exploration of quinoxaline derivatives for antiviral activity is not accidental but a result of a systematic approach, leveraging the knowledge of their chemical properties and prior therapeutic applications. Their mechanisms of action against cancer cells provide valuable insight into how quinoxaline derivatives might be structured to interfere with viral replication or protein function. By drawing parallels between the structural requirements for antitumor and antiviral efficacy, researchers can tailor quinoxaline scaffolds to target specific viral proteins or pathways.^9^

Thus, the dual focus on the expedited redevelopment of existing pharmacophores and the design of novel quinoxaline-based antivirals represents a dynamic and multifaceted response to a global health emergency. By integrating the insights gained from their anticancer potency, the potential of quinoxaline derivatives is being re-envisioned to confront the challenge posed by fast-spreading viral diseases such as COVID-19. In doing so, the same properties that made quinoxaline derivatives valuable in oncology are being repurposed to explore their utility in inhibiting viral entry, replication, and maturation within host cells. The impetus for such investigation is bolstered by the proven success of quinoxaline compounds in antiviral applications, exemplified by the inclusion of quinoxaline moieties in the design of agents like Glecaprevir aimed at combating hepatitis C virus (HCV). The apparent versatility of these molecules propels them to the forefront of antiviral research.^4^

Further exploration of quinoxaline derivatives has revealed their propensity to serve as inhibitors against a spectrum of respiratory viruses, including influenza and various coronaviruses. The malleability of the quinoxaline structure allows for fine-tuning of its pharmacophoric elements, optimizing interactions with viral targets such as enzymes critical for the viral life cycle, structural proteins, and even host cell receptors involved in viral entry. These modifications are informed by structure–activity relationship (SAR) studies, which dissect the functional groups associated with antiviral activity and guide the synthesis of more potent and selective analogues The potential for quinoxaline derivatives to disrupt viral RNA synthesis, or protein assembly presents a compelling case for their inclusion in high-throughput screening assays against SARS-CoV-2, and other emergent pathogens, to identify promising leads for fast-tracked development. It is crucial to leverage the full extent of modern drug development techniques, including computational modeling, virtual screening, and medicinal chemistry, to advance the discovery and optimization of quinoxaline-based antivirals rapidly. In the endeavour to stretch the boundaries of quinoxaline applicability beyond the realm of cancer therapy, meticulous investigation into their antiviral efficacy is being harnessed. Quinoxaline chemistry continues to expand, with ongoing modifications yielding novel derivatives that offer hope for creating versatile and potent pharmaceutical agents. This promise, rooted in robust chemical principles and a deepened understanding of biological interactions, encourages a concerted effort to unlock the full therapeutic potential of quinoxaline derivatives in combating not just present viral adversaries but also future ones.

Quinoxaline derivatives, characterized by their planar polyaromatic structure, are posited to be effective antiviral agents against influenza due to their potential interaction with the NS1 protein, which is an invariant protein encoded by the influenza virus, thus making it a valuable target for therapeutic intervention.^2,14,15^ Additionally, there is a notable interaction between the nucleocapsid protein (N.P.) of the SARS coronavirus and human Cyclophilin A (CypA), in which the N.P. demonstrates a high affinity binding that results in significant inhibition of CypA's activity, indicating a possible avenue for antiviral treatments.^2^ Compounds structured on bicyclic heterocyclic frameworks have been shown to disrupt the interaction between the nucleocapsid protein (N.P.) of HCoV-OC43 and its RNA by binding to the N-terminal domain of the N.P., rendering them potential inhibitors of SARS-related coronaviruses.^16^ Furthermore, computational docking studies have revealed that certain quinoxaline derivatives bind with high affinity to the protease responsible for SARS-CoV-2 replication, thereby indicating their capacity to curb viral propagation.^17^ In the context of COVID-19, which may be interpreted as a syndrome associated with retinoic acid depletion, inhibiting the metabolism of retinoic acid, mainly through its interaction with TLR receptors, emerges as a plausible therapeutic strategy. Synthesized analogs from the heterocyclic classes of imidazo[1,5-*a*]quinoxaline and pyrazolo[1,5-*a*]quinoxaline have been evaluated for their ability to impede the activation of NF-kB *via* modulation of TLR receptors.^5^ Additionally, compounds from the pyrrolo[1,2-*a*]quinoxaline series have been identified as promising candidates for inhibiting the main protease of SARS-CoV-2 and as activators of Sirt6, which are hypothesized to suppress the virus.^6,18^ These findings underscore the therapeutic potential of novel heterocyclic compounds in the ongoing battle against COVID-19.

## 2. Biological activity, synthesis approaches, and structure–activity relationship

### 2.1 Quinoxaline derivatives as potential influenza inhibitors

Influenza viruses, a tripartite group of RNA viruses including types A, B, and C, are known for causing highly infectious respiratory illnesses in humans.^19^ The influenza A viruses are notorious for triggering the most debilitating forms of the disease, as exemplified by the H1N1 strain that incited the flu pandemic of 2009.^20^ Given this backdrop, creating small-molecule therapies targeting influenza is a critical research objective for the current decade. Here, we provide an in-depth analysis of the latest research, outlining new findings, the potential therapeutic mechanisms of quinoxaline derivatives, and their specific implications for treating respiratory viral infections such as COVID-19 and influenza. Precision biophysical methodologies and structural investigations utilizing high-resolution nuclear magnetic resonance (NMR) and X-ray crystallography have elucidated that the NS1A protein's N-terminal domain adopts a distinctive dimeric structure characterized by six helices.^21,22^ Building on this insight, You, L. *et al.* have pioneered the design of quinoxaline derivatives intended to mimic the molecular architecture of epigallocatechin-3-gallate, with substitutions at the 2nd, 3rd, and 6th positions of the quinoxaline scaffold being pivotal (Fig. 4).

Intercalation assays with *in vitro* fluorescence polarization-based binding assays (FP-assays) showed that binding carboxyfluorescein-labeled dsRNA (FAM-dsRNA) to the NS1A protein leads to reduced fluorophore (FAM) mobility, and as a result, the fluorescence polarization rise. Adding possible NS1A inhibitors that target the dsRNA binding domain moved the FAM-dsRNA away from NS1A and lowered the fluorescence polarization. Additionally, an FP-based assay involving probe dsRNA intercalation of the quinoxaline derivatives was used as a control experiment in order to target NS1A rather than dsRNA. The result indicated that the quinoxaline derivatives can disrupt the dsRNA binding to NS1A protein. So intercalation assays with double-stranded RNA (dsRNA) showed analogs 9-a and 9-b inhibit viral functionality by engaging the dsRNA binding domain of NS1A and concluded the effective suppression of influenza A virus proliferation. The SAR analyses have identified bis-2-furyl-modified analogs to exhibit superior antiviral efficacy.

**Fig. 3:**
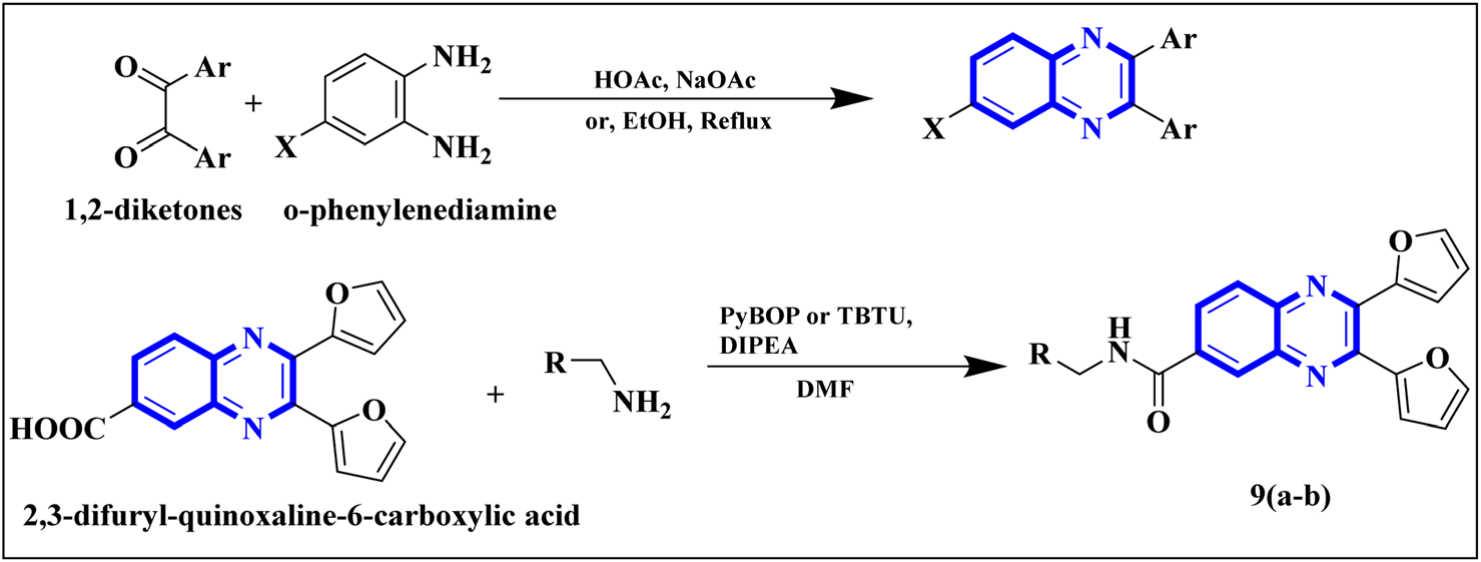
The production of 2,3-bis(furan-2-yl)-6-(3-*N*,*N*-diethyl carbamoyl-piperidine) carbamoyl amino quinoxaline involves a synthetic route that commences with the fusion of 1,2-diketones with derivatives of *o*-phenylenediamine to create substituted quinoxalines. This reaction proceeds under heated conditions in either ethanol or a mixture of acetic acid and sodium acetate. Subsequently, 2,3-difuryl-quinoxaline-6-carboxylic acid is reacted with various amines, with the aid of coupling reagents such as PyBOP or TBTU and DIPEA as the base, yielding a diverse range of amide-functionalized quinoxaline analogs.^14^

Meanwhile, the quinoxaline core has been maintained across variants, with the introduction of various aromatic moieties, including 2-furyl at the 2nd and 3rd positions and an assortment of substituted phenyl groups or heterocycles linked *via* an amide bond at the 6th position, producing compounds of notable potency. The synthetic pathway described leads to the formation of 2,3-di(furan-2-yl)-6-(3-*N*,*N*-diethyl carbamoyl-piperidine)carbonyl amino quinoxaline. This process starts with condensing 1,2-diketones with *ortho*-phenylenediamine derivatives under reflux conditions in either ethanol or a mixture of acetic acid and sodium acetate (Fig. 3). Following this, 2,3-difuryl-quinoxaline-6-carboxylic acid is coupled with a variety of amines employing coupling agents such as PyBOP or TBTU and DIPEA as a base, culminating in a library of amide-modified quinoxaline derivatives.^14^

**Fig. 4:**
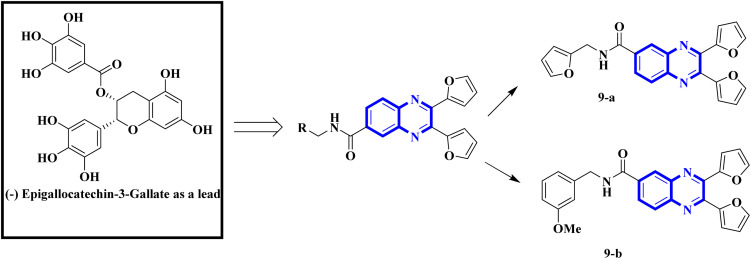
Amide derivatives of quinoxaline based on mimic (−) epigallocatechin-3-gallate novel and SAR of series of quinoxaline-2-mercapto-acetylurea analogues.^14^

**Fig. 5:**
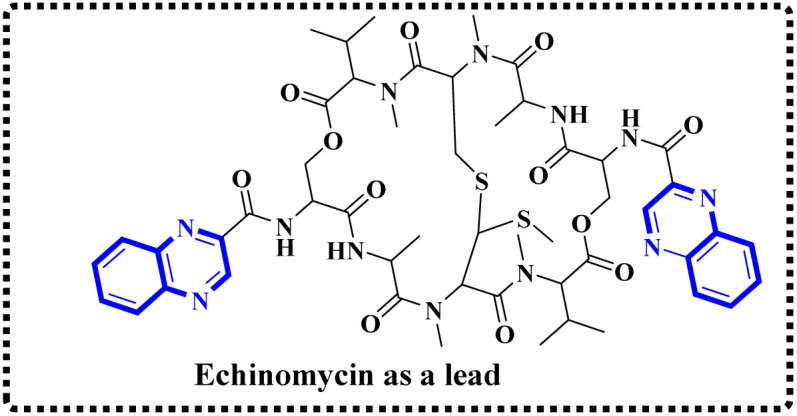
Synthesis pathway of echinomycin derivatives and analogues.

**Fig. 6:**
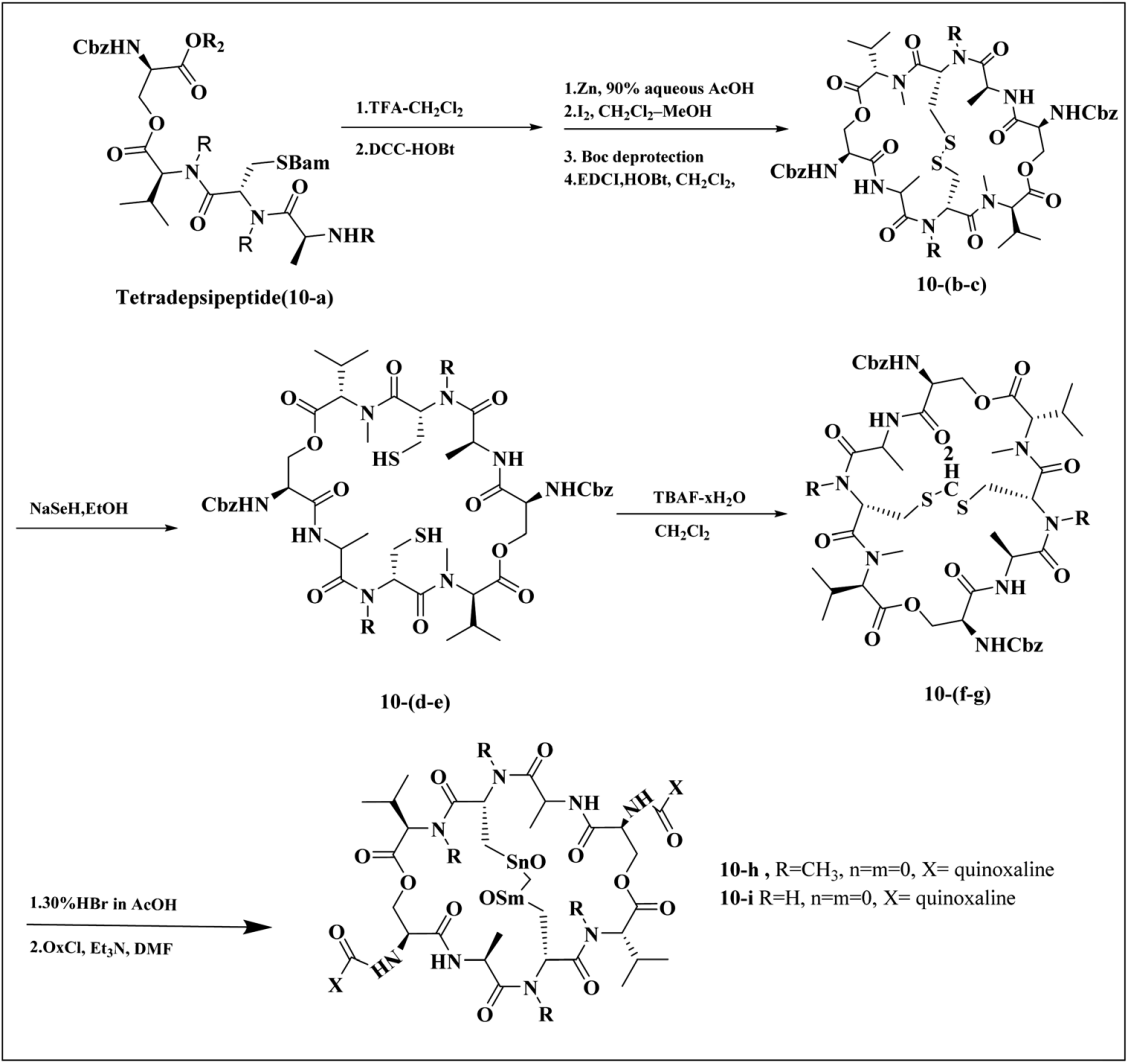
Synthesis pathway of echinomycin derivatives and analogues.^1,23^

Minor, P.D. and associates have documented the antiviral potentials of echinomycin (Fig. 5) against influenza and a range of other viruses. They have crafted various echinomycin derivatives, including formulations such as methyl sulfonium perchlorate (Me-Ech) and the monosulfoxide, disulfoxide, and sulfone versions. Additionally, they generated an echinomycin analog featuring a methylene dithioether group. Reflecting on the exploration of echinomycin derivatives' bioactivities, a new cohort of quinoxaline derivatives, specifically 10h and 10i (Fig. 6), enriched with thioether, sulfoxide, and sulfone functionalities, has been developed.

In a systematic process, the new compounds 10-h and 10-i were synthesized from a cyclic octa depsipeptide framework. The four initial steps were followed by Pa ester deprotection (Zn, 90% aqueous AcOH, at 0 °C, 4 h), disulfide bond formation (disulfide-linkage octadepsipeptide, I2 at 25 °C), Boc deprotection, and cyclization by 1-[3-(dimethylamino)-propyl]-3-ethylcarbodiimide hydrochloride (EDCI). The next step was involved the reduction of disulfide bridges using sodium selenohydride (NaSeH), followed by the incorporation of a methylene group between two *N*-methyl cysteine residues within the S–S bridge; this step was facilitated by using tetra-*n*-butylammonium fluoride (TBAF) in dichloromethane (CH_2_Cl_2_). Subsequent stages included detaching the carboxybenzyl (Cbz) protective group and introducing an acyl group through the reaction with quinoxalyl chloride. The strategic insertion of a methylene moiety where a disulfide bond existed aimed to attenuate the cytotoxicity associated with echinomycin and enhance its analogues' water solubility. Echinomycin has been observed to selectively inhibit the synthesis of viral proteins, including hemagglutinin, neuraminidase, and M protein, at substantially low concentrations without impeding the synthesis of host cellular proteins, even at elevated levels. The study emphasized that the 10-h derivative displays activity against vancomycin-resistant enterococci (VRE) within a minimum inhibitory concentration (MIC) spectrum of 0.5–8.0 mg mL^−1^, in contrast to echinomycin's MIC of 0.25 mg mL^−1^, suggesting that it can exert a potent effect on the influenza virus.^1,23^

**Fig. 7:**
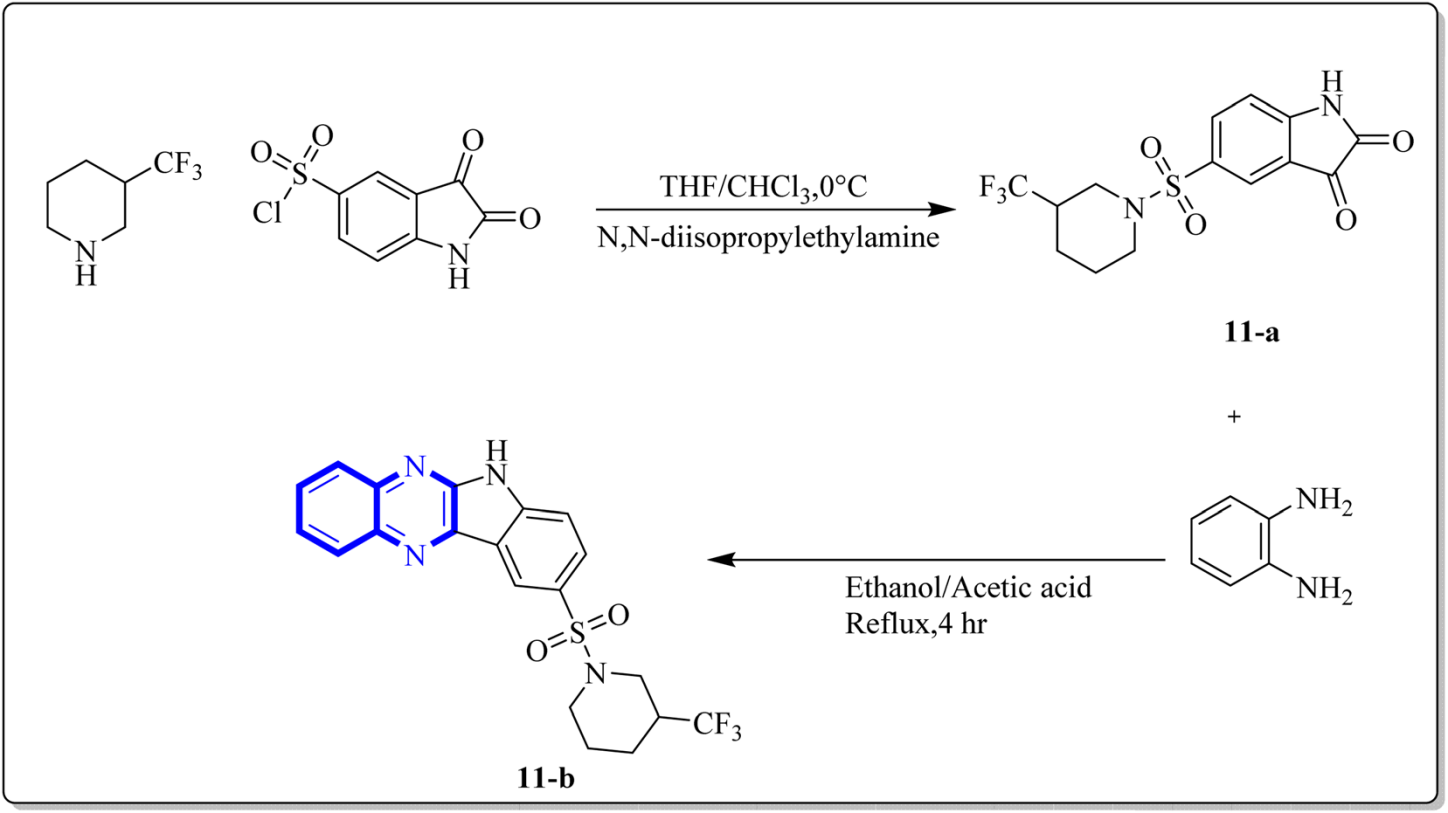
Synthesis pathway of indolo[2,3-*b*]quinoxaline derivatives.^24^

Ezz Eldin, R.R. and co-researchers have demonstrated that newly synthesized derivatives of isatin, including the indolo[2,3-*b*]quinoxaline hybrid, exhibit efficacy against the H1N1 strain of the influenza virus. The safety profile of these compounds was substantiated in non-cancerous cells, with special emphasis on the quinoxaline derivative (11-b), which not only exhibited strong inhibitory activity with an IC_50_ of 0.2164 μM against H1N1 but also displayed minimal toxicity, as evidenced by a significantly high cytotoxicity concentration 50% (CC50) value of 315 578.68 μM which is described as the concentration of a drug or compound that is cytotoxic to 50% of a population of cells. Furthermore, the reduction in viral gene expression, as assessed by quantitative PCR, supported the antiviral activity of the compounds. Computational molecular docking and ADME prediction analyses corroborated the compound's binding efficacy and favourable pharmacokinetic attributes. The synthesis of the indolo[2,3-*b*]quinoxaline hybrid derivative was achieved by the cyclo-condensation of *o*-phenylenediamine. The synthetic pathway outlined for creating this novel isatin hybrid (Fig. 7) began with the formation of trifluoromethyl piperidin-1-ylsulfonyl isatin (11-a), which was synthesized by reacting chlorosulfonyl isatin with trifluoromethylpiperidine. Isatin derivatives, including an indolo[2,3-*b*]quinoxaline hybrid, demonstrate antiviral capabilities against the H1N1 strain of influenza virus. these compounds proved reliably safe for normal cells, with a special mention of the quinoxaline-based molecule (11-b). This compound exhibited a IC_50_ of 0.2164 μM against H1N1, owing to a substantially higher selectivity index, indicated by a CC50 value of 315, 578.68 μM, denoting minimal toxicity. Quantitative polymerase chain reaction (qPCR) assays that reported a reduction in viral gene expression further substantiated the antiviral efficacy. Computational molecular docking and absorption, distribution, metabolism, and excretion (ADME) studies of this compound showed promising binding affinity and pharmacokinetic profiles. The synthetic process which yielded the indolo[2,3-*b*]quinoxaline hybrid involved a cyclo-condensation step with *o*-phenylenediamine. The initial step in fabricating the novel hybrid molecule involved synthesizing trifluoromethyl piperidin-1-ylsulfonyl isatin (11-a) through a reaction between chlorosulfonyl isatin and trifluoromethylpiperidine. Subsequently, the indolo[2,3-*b*]quinoxaline was synthesized by condensing trifluoromethyl piperidin-1-ylsulfonyl isatin (11-a) with *o*-phenylenediamine.^24,25^

### 2.2 Quinoxaline derivatives as potential anti-SARS coronavirus inhibitors

**Fig. 8:**
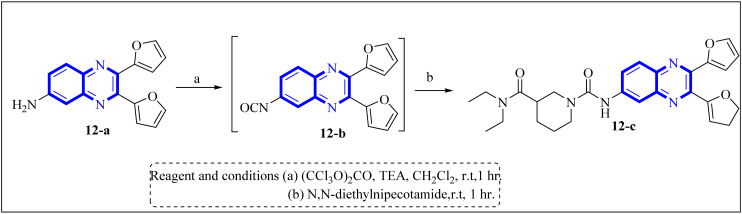
Carbonylamino quinoxalines were suggested as a potent inhibitor against human Cyclophilin A.^2^

Li, J., and colleagues proposed a new quinoxaline compound, designated as 2,3-di(furan-2-yl)-6-(3-*N*,*N*-diethyl carbamoyl-piperidine)carbonyl amino quinoxaline or compound 12-c, as a potent inhibitor of human Cyclophilin A (CypA) in mouse spleen cell proliferation. CypA is a widely present cellular enzyme that was shown to have a high binding affinity to the nucleocapsid protein (N.P.) of SARS-CoV. The research showcased that through sequence alignment and molecular modeling, the Trp302-Pro310 loop of the SARS-CoV N.P. was compatible with the active site of CypA, facilitated by various interactions, including hydrogen bonds and cation–π and –CH–π hydrogen bonding. Therefore, the hydrophobic interaction between CypA/compound 12-c might be able to inhibit the PPIase activity of CypA. The inhibitor's effectiveness in CypA PPIase activity was quantified, with an IC_50_ value reported at 0.41 μM, demonstrating compatibility with the hydrophilic carboxymethylated dextran matrix utilized in a CM5 sensor chip (Biacore). Fluorescence titration further supported the claim that compound 12-c is a strong contender for CypA inhibition. Synthesis of compound 12-c followed a sequential methodology; initially, 6-amino-2,3-di(furan-2-yl)quinoxaline (12-a) was reacted with triphosgene in the presence of triethylamine under a nitrogen atmosphere at room temperature, giving rise to 2,3-di(furan-2-yl)-6-isocyanate quinoxaline (12-b). The final step involved reacting *N*,*N*-diethylnipecotamide with compound 12-b to produce compound 12-c (Fig. 8). Reactions were generally conducted in round-bottomed flasks that had been dried in an oven under a protective nitrogen atmosphere while stirring was provided by a magnetic stirrer. Purification of reagents included distillation of triethylamine over sodium and dichloromethane over calcium hydride (CaH_2_).^2^

**Fig. 9:**
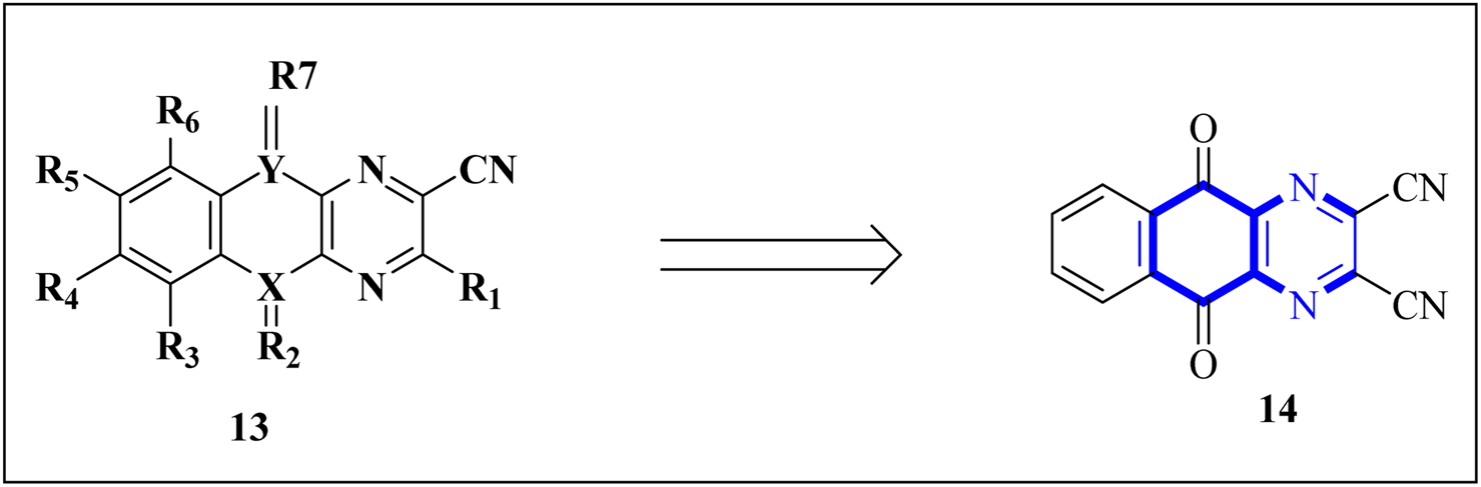
5,10-Dioxo-5,10-dihydro-benzo[*g*]quinoxaline-2,3-dicarbonitrile.^26^

A novel structure of the anti-SARS coronavirus effect based on a quinoxaline derivative was reported by al G.e. (Fig. 9). They studied De-ubiquitination enzymes, including ubiquitin-specific protease (USPs), and the ubiquitin pathway regulates protein degradation.^26^

**Fig. 10:**
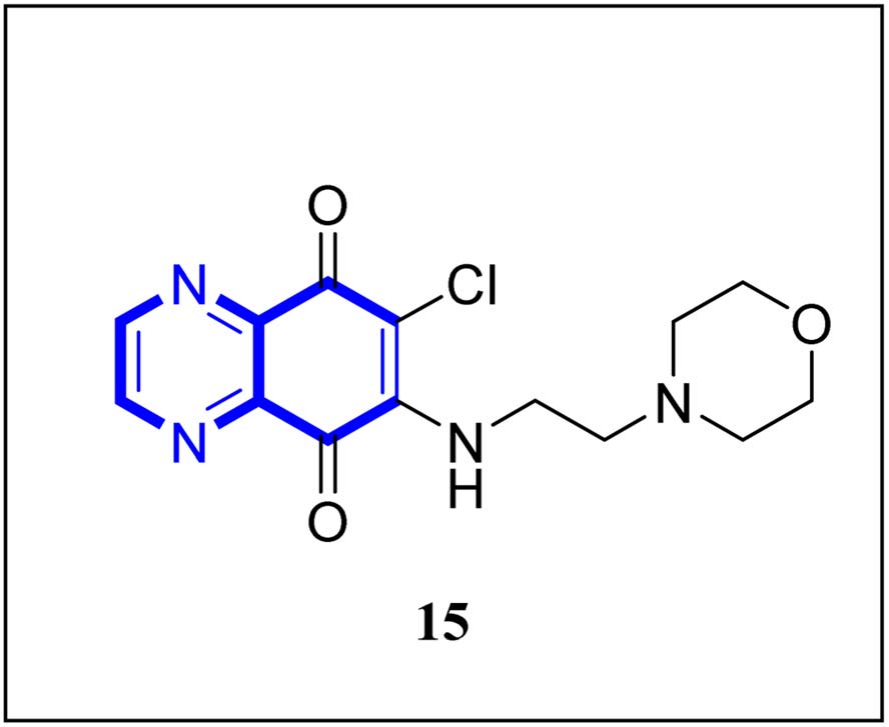
6-Chloro-7-(2-morpholin-4-yl-ethylamino)quinoxaline-5,8-dione compound as a coronavirus replication inhibitor.^16^

Research has indicated the potential role of coronavirus nucleocapsid proteins (N.P.) as targets for antiviral drugs. In their study, Chung-ke Chang and colleagues have derived agents that target the N.P., such as compound 15 employing *in silico* virtual screening methods aimed at coronavirus nucleocapsid proteins. They used experimental approaches to corroborate their findings, including surface plasmon resonance (SPR) assays by the repeated intergenic sequence of HCoV-OC43, 5′-biotin-(UCUAAAC)4-3′, as a probe, X-ray crystallography, and molecular docking studies. Among the identified compounds, the quinoxaline-based compound in SPR assay notably reduced the RNA-binding ability of the N.P. by over 20%. Specifically, it was very effective in inhibiting the RNA-binding activity of N.P.'s N-terminal domains (N-NTDs) from HCoV-OC43 *in vitro*. X-ray crystallographic analysis revealed that the interaction of the quinoxaline molecule with the N-terminal domain impedes the RNA association with HCoV-OC47 NP (Fig. 10).^16^

### 2.3 Quinoxaline derivatives as potential anti-SARS-CO-2 coronavirus

**Fig. 11:**
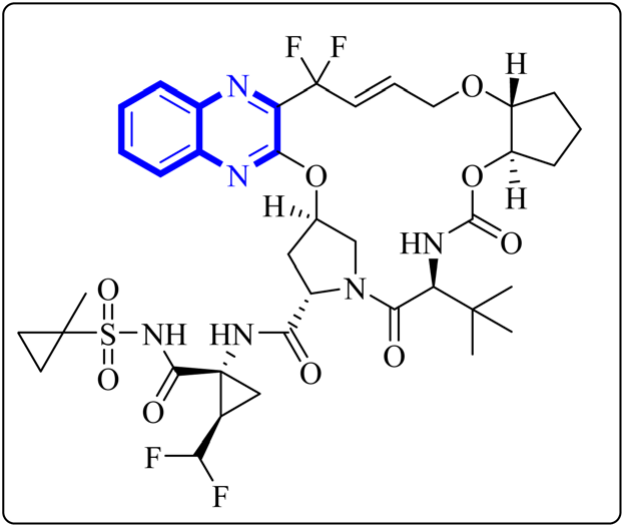
Glecaprevir structure.^4^

A thorough molecular dynamics (M.D.) simulation study evaluated the binding of various drugs to the main protease (Mpro) of SARS-CoV-2, including their combinations. The study considered anti-HCV drugs, namely Elbasvir and Glecaprevir, alongside the anti-HIV medication Ritonavir, as potential frameworks for the experimental testing and pharmacophore development of anti-COVID-19 therapeutics. Such a strategy is pivotal for creating drugs that are less susceptible to viral mutations and specifically target the Mpro enzyme of SARS-CoV-2. Glecaprevir, a quinoxaline-based molecule shown in Fig. 11, is known as an inhibitor of the nonstructural (N.S.) protein protease of hepatitis C virus (HCV). Owing to the structural similarities between the proteases of SARS-CoV-2, HCV, and HIV, it is hypothesized that medications designed to inhibit HCV and HIV could also yield positive therapeutic effects against SARS-CoV-2. The study further evaluated the interaction of Glecaprevir with the catalytic site and two novel allosteric sites of Mpro. According to Bhat, Z.A., and associates, the quinoxaline ring of Glecaprevir formed fundamental interactions predominantly with several hydrophobic amino acid residues, including Leu286, Leu287, Leu272, Met276, Ala285, Gly275, and Gly278. Additionally, a significant polar interaction was observed with the side chain of Asn277 situated within the α-helical domain of Mpro. The team also discovered potential druggable allosteric sites located at the intra-domain and dimeric interface of the enzyme, providing insights into novel therapeutic targets.^4^

**Fig. 12:**
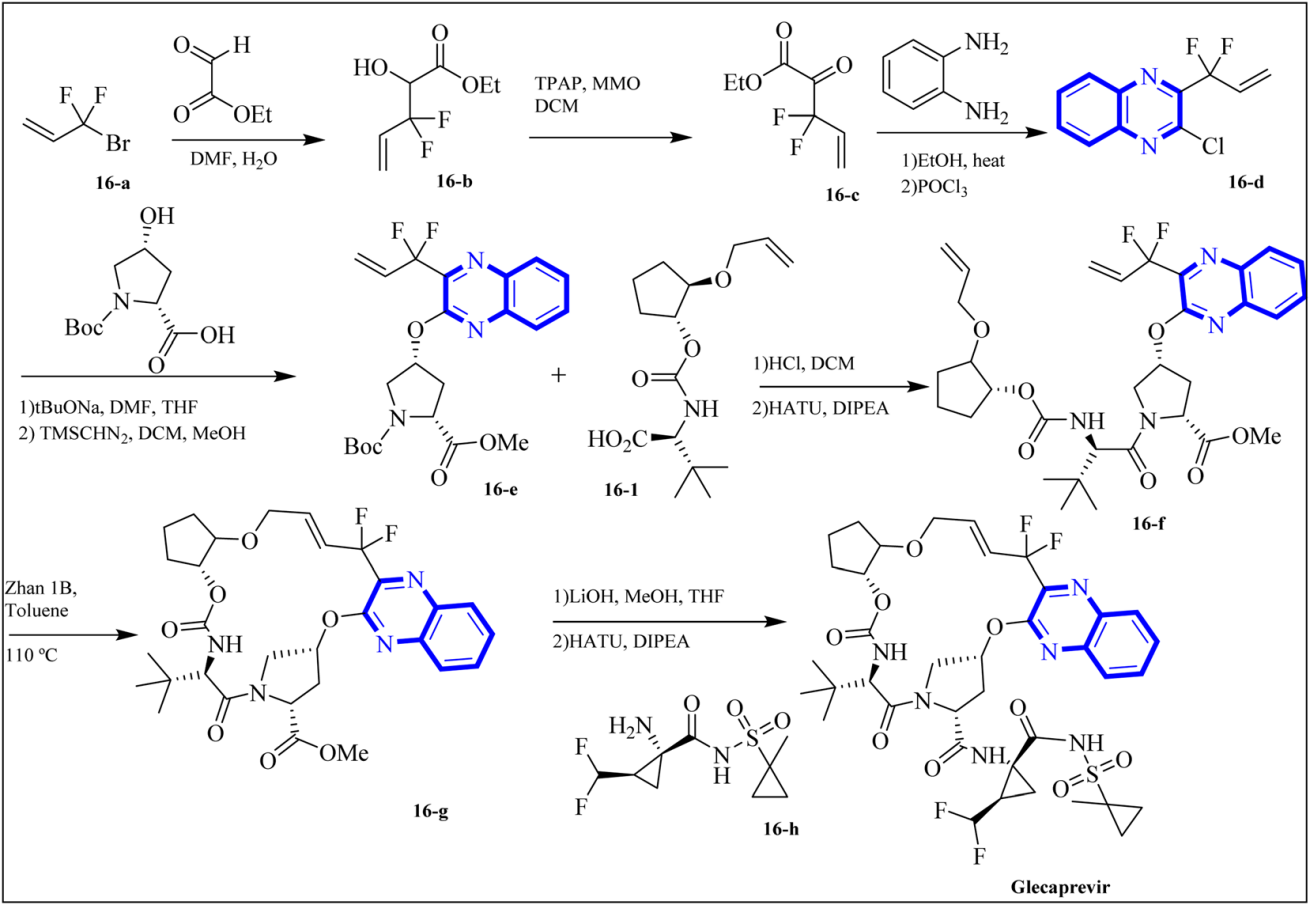
Synthesis pathways of Glecaprevir.^4^

The following describes the process of synthesizing glecaprevir, beginning with building the linker region, intermediate 16-1 (Fig. 12). Diacetate intermediate produced by acetylating racemic *trans*-1,2-cyclopentanediol accomplished in a lipase buffer to create (1*R*,2*R*)-2-hydroxycyclopentyl acetate. The preparation (1*R*,2*R*)-2-(allyloxy) cyclopentane-1-ol involved the creation of allyl ether in the presence of sodium hydride and allyl bromide in DMF. Intermediate 16-1 was formed by reacting (1*R*,2*R*)-2-(allyloxy) cyclopentane-1-ol with (*S*)-2-amino-3,3-dimethylbutanoic acid and activating it with phosgene. Difluoroallyl bromide compound (16-a) and ethyl glyoxalate performed a Barbier reaction with indium to generate difluoro alcohol (16-b), which TPAP/NMO then oxidized to produce keto ester (16-c). The intermediate 16-d for chloroquinoxaline was prepared by condensing 16-c with *o*-phenylenediamine in ethanol after being treated with POCl_3_. Under basic procedures, coupling of 16-d with L-N-Boc-4-hydroxyl-proline occurred without any problems, and the intermediate esterified to produce ester 16-e. Diene 16-f synthesized by Boc deprotection, amide coupling, and intermediate 16-1. The ring-closing metathesis of diene 16-f in toluene could successfully produce the macrocyclic intermediate 16-g. Eventually, glecaprevir was synthesized by saponifying intermediate 16-g and linking it to amine 16-h. There was a procedure by which the quinoxaline fragment of glecaprevir was created.^4,27^

**Fig. 13:**
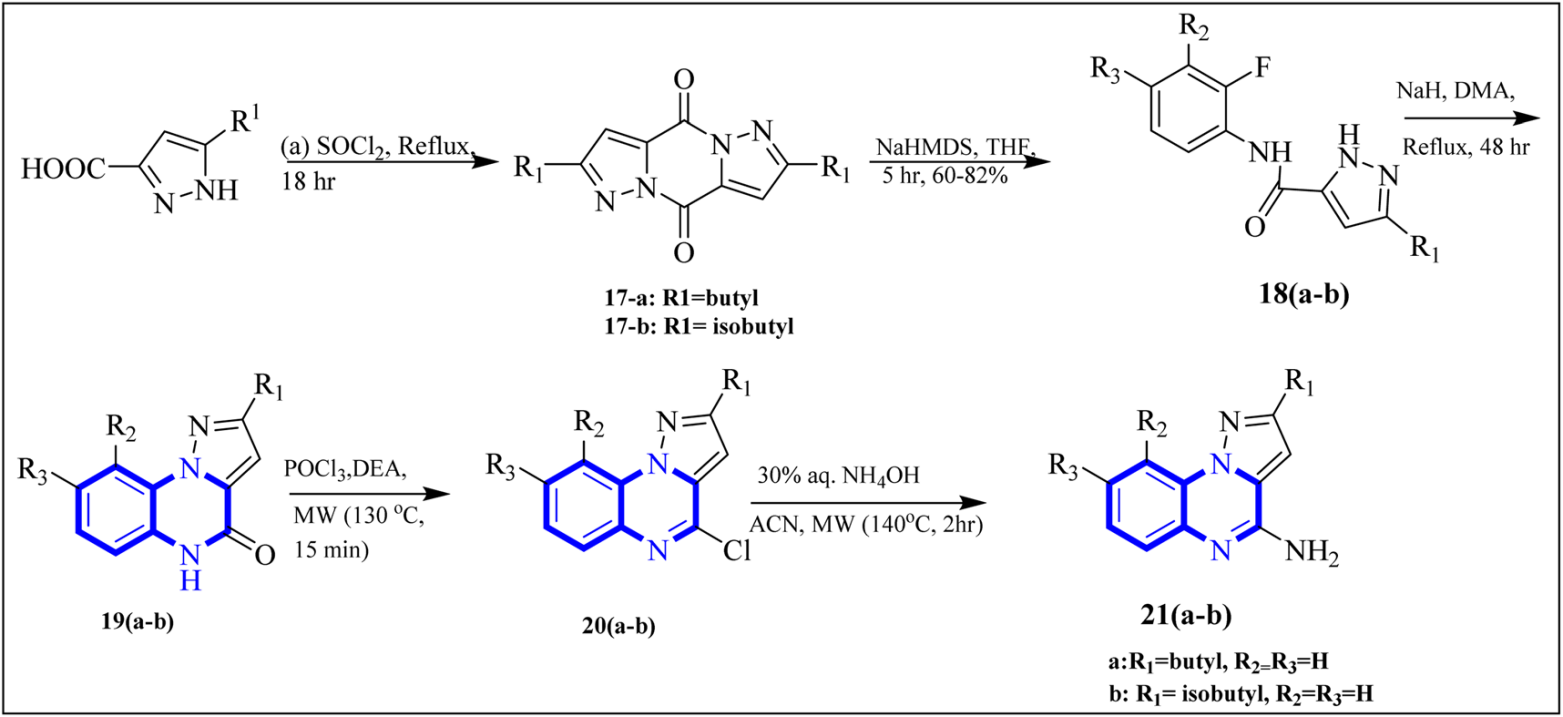
Synthesis rutes of pyrazolo[1,5-*a*]quinoxaline derivatives.^5^

COVID-19 has been associated with a condition known as retinoic acid depletion syndrome, similar to other inflammatory diseases. Substances that impede the liver cytochrome P450 oxidase system are deemed potential COVID-19 therapeutics as they prevent retinoic acid metabolism. A common feature of numerous relevant infectious and autoimmune conditions is chronic immune activation, which often results from overstimulation of toll-like receptors (TLRs), and this has spurred the development of TLR antagonists. When retinoic acids are depleted, this can lead to a dominance of the NF-κB pathway, resulting in a surge of cytokine production, a state referred to as “retinoic acid depletion syndrome”.^28^ Bou Karroum, N *et al.* explored a range of synthesized compounds, specifically derivatives of the heterocyclic imidazo[1,5-*a*]quinoxaline and pyrazolo[1,5-*a*]quinoxaline categories, for their ability to restrain NF-κB translocation within HEK-Blue cells that overexpress TLR7 or TLR8. Pyrazolo[1,5-*a*] synthesized quinoxaline derivatives followed the delineated procedures (Fig. 13). Here, dimers 17(a-b) were generated from the bimolecular condensation of 5-substituted 1*H*-pyrazole-3-carboxylic acids with thionyl chloride. Subsequent coupling of 17(a-b) with fluoro aniline derivatives yielded intermediates 18(a-b). The formation of tricyclic structures, 19(a-b), was achieved through intramolecular cyclization of 18(a-b) under fundamental conditions. The 19(a-b) reaction with phosphorus oxychloride and *N*,*N*-diethylaniline produced 20(a-b). The final compounds, 21(a-b), were obtained by replacing the chlorine atom with aqueous ammonia in 20(a-b).

**Fig. 14:**
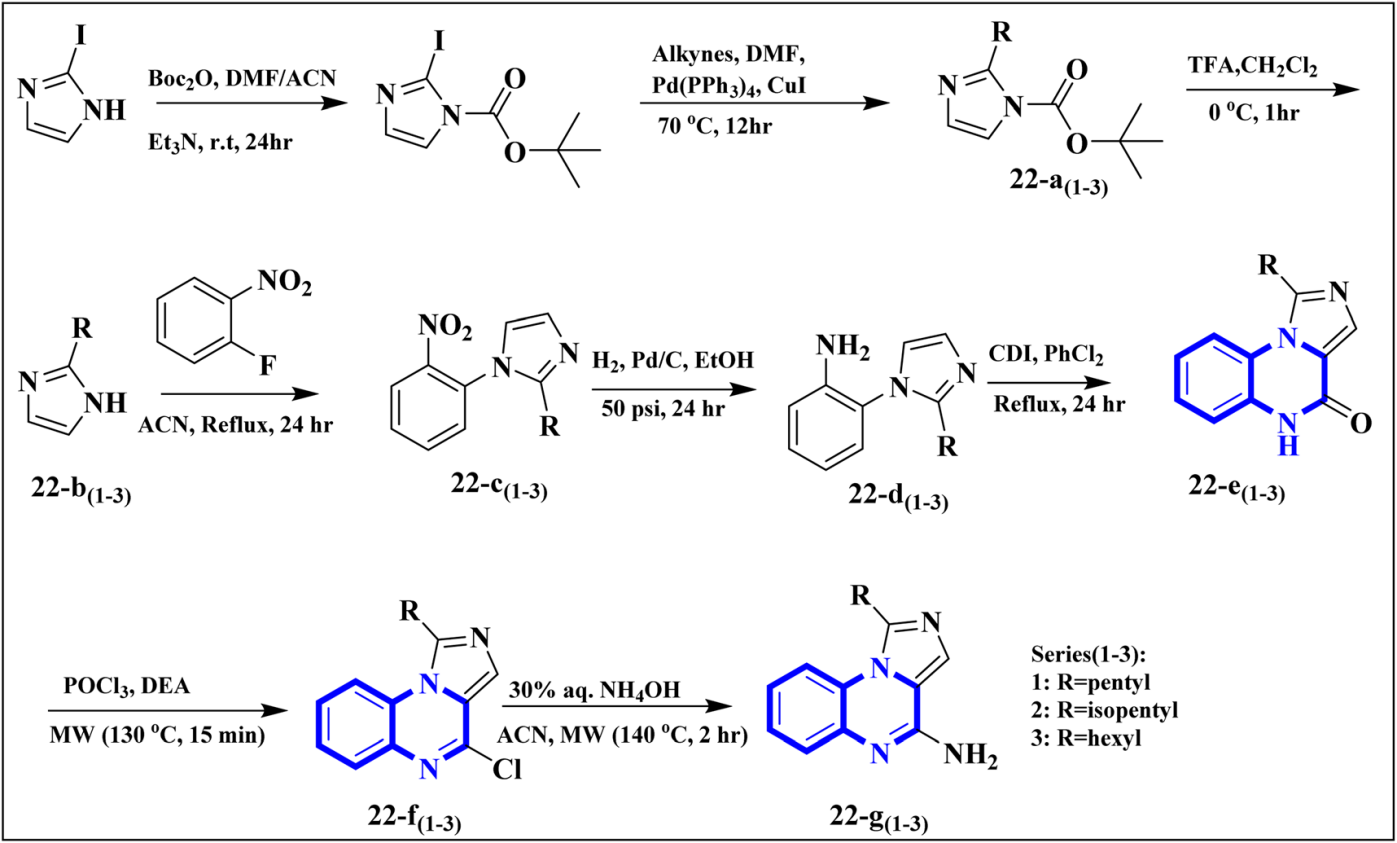
Synthesis rutes of imidazo[1,5-*a*]quinoxaline derivatives.^5^

The research team also generated imidazo[1,5-*a*]quinoxaline derivatives, integrating diverse alkyl chain lengths at the 1-position on the imidazole moiety. Firstly, protective measures for the amino functionality were undertaken on the 2-iodo-1*H*-imidazole starting material, paving the way for the generation of alkylated imidazole compounds, denoted as the 22-a (1-3) series, *via* Sonogashira cross-coupling reactions. Subsequent removal of the protective group enabled the attachment of these imidazoles (22-b (1-3) series) to *ortho*-fluoronitrobenzene through a coupling process facilitated by an abundance of potassium carbonate, producing the 22-c (1-3) series of compounds. The next phase entailed a two-step reduction: one to transform the nitro group and another to address the alkyne functionality. This set the stage for constructing the 22-e (1-3) series based on intramolecular cyclization of the precursors 22-d (1-3). The catalyst employed during this cyclization step was carbonyldiimidazole. Subsequently, the nucleophilic displacement utilizing aqueous ammonia and chlorination mediated by phosphorus oxychloride afforded the 22-f (1-3) series. In a particular synthesis pathway outlined in the stated synthetic scheme (Fig. 14), the 22-g (1-3) series was prepared, featuring a butyl chain attached to the imidazole ring. The SAR assessment of these imidazo[1,5-*a*]quinoxaline entities revealed a moderate elevation in their antagonistic efficacy when the alkyl chains consisted of 4 to 5 carbon atoms (butyl, pentyl, and isopentyl groups). However, extending the alkyl chain length beyond five carbon atoms to hexyl led to diminished antagonistic activity.

In a concentration of 15 μM, derivatives of pyrazolo[1,5-*a*]quinoxaline achieved an inhibition rate near 50% for NF-κB translocation in HEK-blue cells with an overexpression of TLR7. These derivatives are recognized as potent and selective TLR7 antagonists, positioning them as promising points of departure for the synthesis of new immunomodulatory compounds with therapeutic potential. Notably, 21-a and 21-b, which possess butyl and isobutyl chains, demonstrated substantial selectivity and potency as TLR7 antagonists, with IC50 values of 8.2 μM and 10.0 μM, respectively. Regarding the SAR analysis, the derivatives from the pyrazolo[1,5-*a*]quinoxaline series that incorporated either butyl or isobutyl tails exhibited the highest antagonistic effect against TLR7. The addition of a methyl group at the 8th position in these compounds was observed to have a negligible impact on their activity.

Conversely, this antagonistic effect was compromised when substituting a methyl group at the 9th position. Furthermore, comparative ligand-docking studies have illuminated a distinctive binding mode for these heterocyclic molecules to a newly identified antagonist binding site for TLR7. These findings, combined with the SAR data, rationalize the potential investigation of quinoxaline derivatives as viable options in the treatment of COVID-19.^5^

**Fig. 15:**
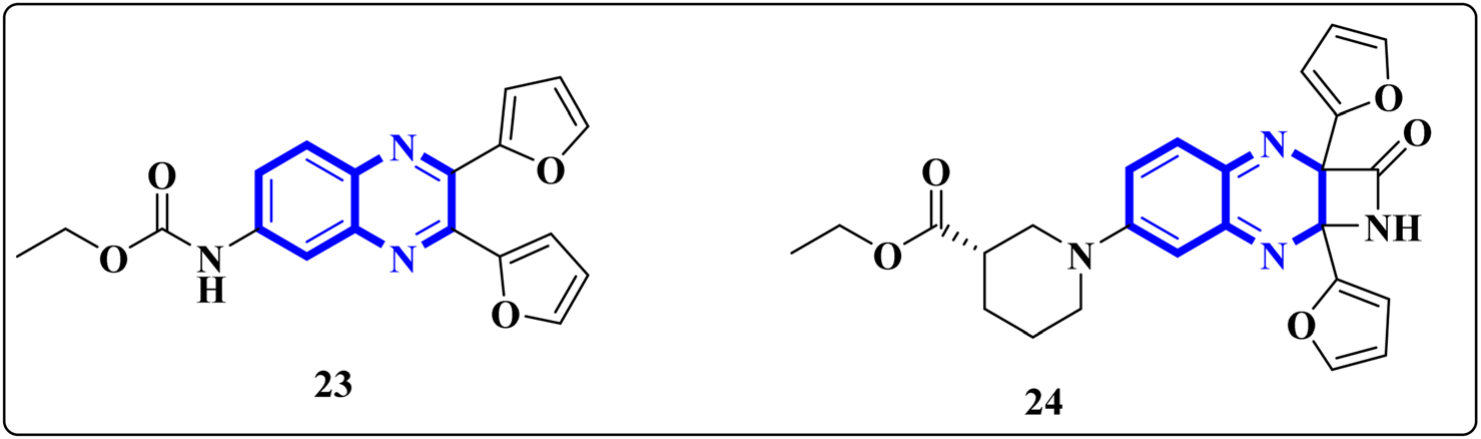
The compounds labeled as 23 and 24, specifically 2,3-di(furan-2-yl)-6-ethoxycarbonylamino quinoxaline and ethyl(3*S*)-1-(2a,8a-di(furan-2-yl)-2-oxo-1,2,2a,8a-tetrahydroazeto[2,3-*b*]quinoxalin-6-yl)piperidine-3-carboxylate, respectively, have been identified as potential agents against SARS-CoV-2. Compound 23 integrates two furan rings at the 2 and 3 positions and an ethoxycarbonylamino group at the 6 position on a quinoxaline scaffold. Compound 24 features a similar furan-quinoxaline structure, with the addition of an ethyl piperidine carboxylate moiety, imparting a distinct stereochemistry represented by the (3*S*) configuration. These molecular components are the focus of anti-SARS-CoV-2 pharmaceutical investigation.^29^

El-Hoshoudy, A. utilized a computational inquiry to investigate the inhibitory impacts of certain quinoxaline derivatives, among other ligands, through molecular docking techniques focused on the 6YB7 protease associated with COVID-19. The main virus protease was docked with some antiviral drugs and natural inhibitory ligands against COVID-19 which were improved for *Escherichia coli* BL21 (DE3) to identify the antiviral activity of these candidates against COVID-19. The findings from the docking studies revealed that quinoxaline derivatives 23 and 24 (Fig. 15) demonstrated significant binding affinity toward the protease, suggesting their capability to suppress the protease's function and consequently mitigate viral infection by COVID-19.^29^

**Fig. 16:**
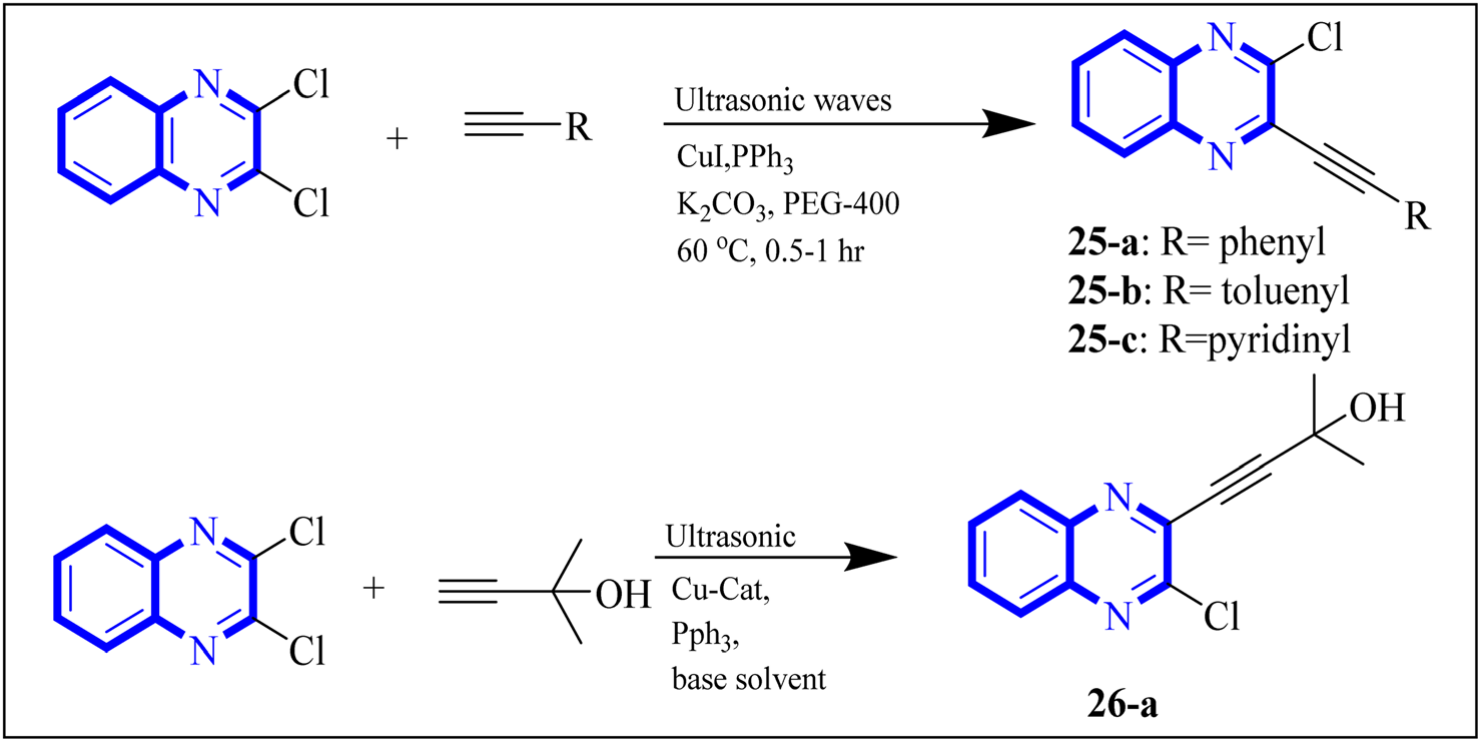
Synthesis of 3-alkynyl substituted 2-chloroquinoxalines facilitated by ultrasonication and catalyzed by copper.^3^

In their research, Shahinshavali, S. *et al.* conducted a sequential and structural analysis of the N-terminal RNA-binding domain (NTD) of N-protein of SARS-CoV-2 in comparison to that of HCoV-OC43, intending to identify shared regions between the two proteins, and active site residues were pinpointed. Drugs such as chloroquine, favipiravir, and quinoline, currently under consideration for their efficacy against coronaviruses, served as the basis for *in silico* docking studies to explore their potential as SARS-CoV-2 inhibitors. The study introduced a novel molecular framework, designated template (D), which incorporated the chloro functional group and pyrazine ring found in the antiviral agent's quinoline and favipiravir. This template, specifically a 3-alkynyl substituted 2-chloroquinoxaline structure, was utilized to evaluate its binding affinity to the N protein's nucleotide binding site of SARS-CoV-2 through molecular docking. The results indicated that template (D) derivatives generally demonstrated decent to moderate binding affinities, all exhibiting values above 5.0 kcal mol^−1^. This novel compound, 3-alkynyl substituted 2-chloroquinoxaline labeled as compound 25(a–c), was synthesized using a more eco-friendly and expedient approach that omitted bi-metallic salt catalysts. It employed a copper-catalyzed C≡C bond formation reaction aided by ultrasonication. The synthesis method produced the compound 26-a efficiently by coupling 2,3-dichloroquinoxaline with readily available terminal alkynes in the presence of CuI, PPh_3_, and K_2_CO_3_ within a PEG-400 medium^30^ (Fig. 16).

In the referenced article, it was determined that most examined compounds exhibited binding affinities ranging from good to moderate when assessed by *in silico* protein interaction studies. The characteristics and magnitude of the substituent groups attached to the alkyne, apart from the chloroquinoxaline portion, significantly influenced their capacity to bind *in silico* with the target protein. SAR analysis inferred that substituents such as hydroxy cycloalkyl, aryl, and heteroaryl at the R position of compounds 25(a–c) exhibited the highest efficacy. Substituents like hydroxy dimethyl, pentyl, or hexyl chains provided moderate efficacy, while hydroxy methylene groups showed lesser efficacy among the substitutions examined. Larger alkyl chains, such as butyl or *tert*-butyl groups, were found to diminish binding affinity further. The chloro functional group was notably engaged in hydrophobic interactions, particularly affecting the binding affinities to the N-terminal RNA-binding domain (NTD) of the N-protein of SARS-CoV-2. The quinoxaline backbone facilitated interactions such as π–π stacking, hydrophobic, van der Waals contacts, and π–cation interactions at the active site. In the realm of SARS-CoV-2 main protease (Mpro) inhibition, novel potent lead compounds have been identified with computer-aided drug design. SARS-CoV-2, characterized as a positive-sense, single-stranded enveloped RNA virus, encodes four structural proteins in two polyproteins: pp1ab and pp1a. The virus-specific chymotrypsin-like protease (Mpro) and papain-like protease (PLpro) are further processed into functional proteins.^3^

**Fig. 17:**
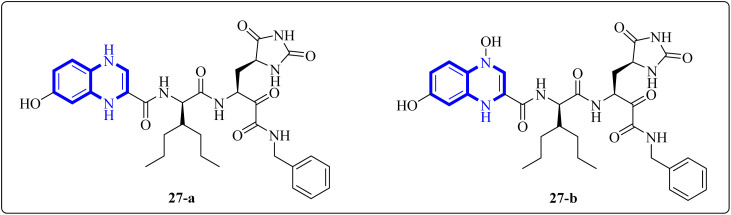
1,4-Dihydroquinoxaline (27-a) and quinoxaline-1(4*H*)-ol (27-b) as peptidomimetic inhibitors of SARS-CoV-2 main protease.^17^

Further enhancing this effort, Frecer, V. and S. Miertus introduced rigid condensed aromatic systems to increase the solvent-exposed surface area of the P3 moiety, enhancing inhibitor binding the binding affinity of the amino acids within the main protease 3CL^pro^ of COVID-19 *via* a pronounced hydrophobic effect. For instance, aromatic compounds like quinoxaline-1(4*H*)-ol (27-b) were chosen for the P3 residue of SARS-CoV-2 Mpro to add an extra hydrogen bond (H.B.) from hetero atoms or functional groups to the side chain of Gln189 in Mpro. Similarly, 1,4-dihydroquinoxaline (27-a) positioned at P3 could donate an additional H.B. to the backbone carbonyl of Glu166. Quinoxaline-1(4*H*)-ol (27-b) was also considered for inclusion as a condensed aromatic moiety at the P3 residue (Fig. 17).^17^

Compound 31, a 4-(5-nitro-thiophen-2-yl)-pyrrolo[1,2-*a*]quinoxaline derivative, has been identified by Divya, K. and colleagues as a potential inhibitor of the main protease of SARS-CoV-268058. In the work of Divya, K. *et al.*, they identified compound 28, or 4-(5-nitro-thiophen-2-yl)-pyrrolo[1,2-*a*]quinoxaline, as a potential inhibitor of SARS-CoV-2's main protease through *in silico* analysis. By conducting a docking study to ascertain the compound's affinity for binding to amino acid residues at the 3CL^pro^ active site of SARS-CoV-2, the research demonstrated the significance of compound 28's interaction. The molecular docking results illustrated that compound 28 establishes noteworthy interactions, predominantly through hydrogen bonds and hydrophobic forces, with residues at the 3CL^pro^ active site this enzyme is crucial to the virus's life cycle. Compound 28's affinity was comparable to established COVID-19 therapeutics like remdesivir and favipiravir.

**Fig. 18:**
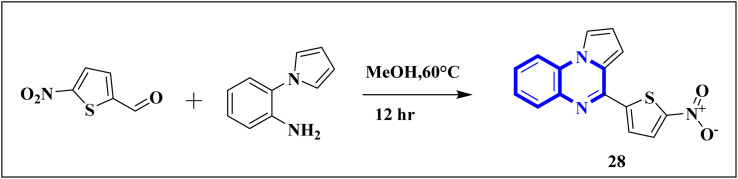
Synthesis of 4-(5-nitro-thiophen-2-yl)-pyrrolo[1,2-*a*]quinoxaline, compound 28.

Synthesis of compound 28 (Fig. 18) involved a direct and simple, catalyst-free process that combined 1-(2-aminophenyl)pyrrole with 5-nitro-2-thiophene carboxaldehyde. This mixture was then subjected to reflux in an oil bath maintained at 60 °C for twelve hours, resulting in a successful yield.^6^

**Fig. 19:**
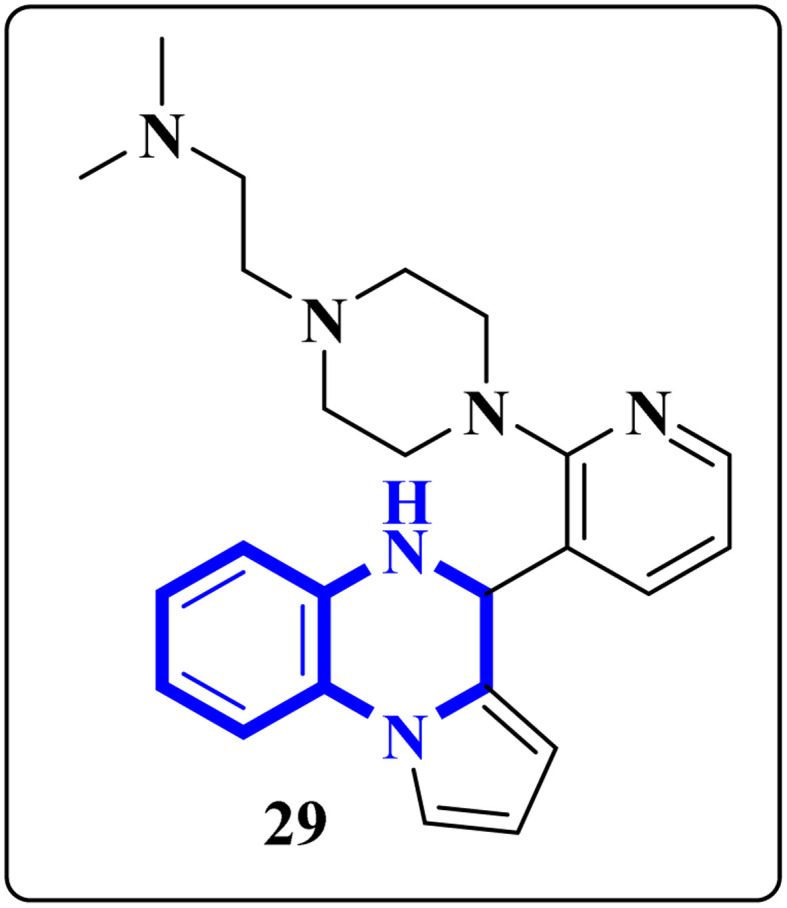
Structure of pyrrolo[1,2-*a*]quinoxaline-based derivatives.^18^

The enzyme group known as sirtuins, specifically the Sirt family, is characterized by its dependence on NAD+ for function. Significant interest has been directed toward activating Sirt6, a member of this family, due to its potential as a therapeutic target for various diseases, including COVID-19. In their study, Xu, J., and colleagues have discovered several new derivatives of pyrrolo[1,2-*a*]quinoxaline (Fig. 19), which act as potent, selective activators of Sirt6. Among these, the compound 2-(4-(3-(4,5-dihydropyrrolo[1,2-*a*]quinoxaline-4-yl)pyridine-2-yl)piperazine-1-yl)-*N*,*N*-dimethylethan-1-amine, referred to as compound 29, stands out for its notably enhanced effectiveness and minimal cytotoxicity. Molecular docking research underscores that compound 29's side-chain protonated nitrogen engages in π–cation interactions with the Trp188 residue, resulting in increased binding within the extended pocket. The study's conclusions highlight that compound 29 in Cell viability assay (A549-hACE2 cells) suppresses SARS-CoV-2 infection, demonstrating an EC_50_ value of 9.3 μM in present of the lead compound UBCS039 for evaluating their potentials against SARS-CoV-2.^18^

**Fig. 20:**
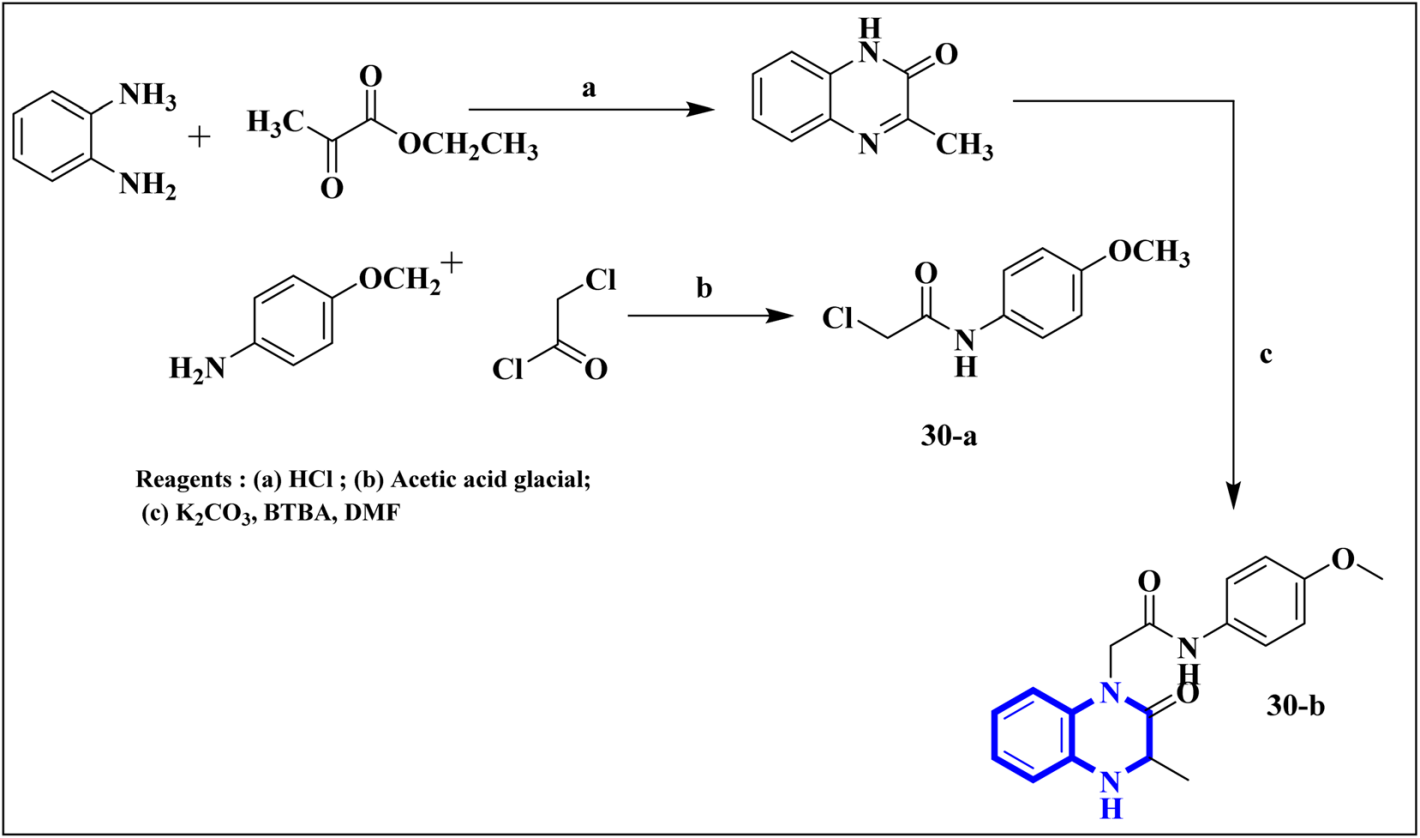
Synthetic route *N*-(4-methoxyphenyl)-2-(3-methyl-2-oxo-3,4-dihydroquinoxalin-1(2*H*)-yl)acetamide, compound 30-b.^30^

Missioui, M., and colleagues have developed a novel quinoxalin compound, named *N*-(4-methoxyphenyl)-2-(3-methyl-2-oxo-3,4-dihydroquinoxalin-1(2*H*)-yl)acetamide (NMPOQAa), designated as compound 30-b. This compound was synthesized and evaluated for potential anti-COVID-19 activity. While the measured experimental parameters were closely aligned with theoretical predictions, a discrepancy was observed in the geometrical alignment owing to differences in torsion angles between the theoretical and experimental structures. Through molecular docking studies, compound 30-b exhibited a behavior consistent with that of the known COVID-19 therapeutic, Remdesivir against the 6M03 protease (a COVID main protease). The SAR study indicated that the NMPOQAa compound by many hydrophobic interactions intracted with the amino acid residues of the active such as, Phe8, Gln100, Arg105, Asp150 and Asp295. The synthetic pathway for compound 30-b began with the condensation of *o*-phenylenediamine with ethyl pyruvate in an aqueous HCl solution, which was maintained at room temperature for half an hour. Subsequently, P-Toluidine was dissolved in glacial acetic acid and cooled in an ice bath. Chloroacetyl chloride was then cautiously added to the solution in increments, with continuous stirring. After completing this reaction phase, adding a sodium acetate resolution precipitated solids within 30 minutes. For the final step, 3-methylquinoxalin-2(1*H*)-one was dissolved in dimethylformamide and combined with 2-chloro-*N*-(4-methyl-2-nitrophenyl)acetamide, a precursor labeled as compound 30-a. This was followed by the introduction of potassium bicarbonate as the base for proton abstraction and a small quantity of benzyl triethylammonium chloride (BTBA) for phase transfer catalysis. The mixture then underwent reflux while being stirred continuously for two hours at 80 °C (Fig. 20).^30^

**Fig. 21:**
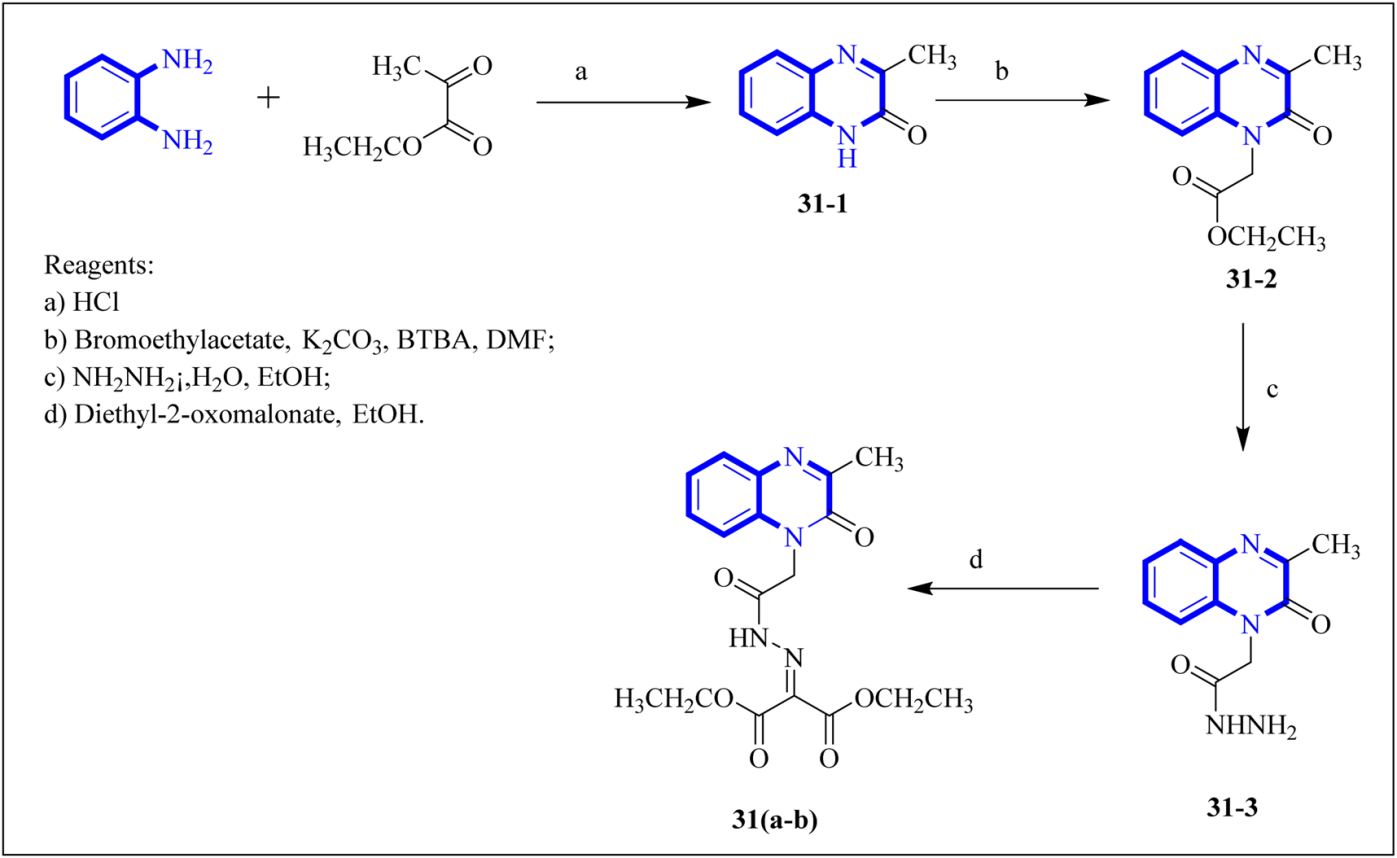
Synthetic route 2-(2-(2-(3-methyl-2-oxoquinoxalin-1(2*H*)-yl)acetyl)hydrazono)malonate, 31(a-b).^31^

Missioui, M. and associates synthesized a novel compound labeled as diethyl 2-(2-(2-(3-methyl-2-oxoquinoxalin-1(2*H*)-yl)acetyl)hydrazono)malonate (MQOAHM), known as compound 31(a-b), to evaluate its potential as a COVID-19 therapeutic. Although initial results did not demonstrate a definitive effect against COVID-19 (MPro, PDB 7BQY), the intricate network of multi-hydrogen bonds and hydrophobic interaction between the newly formed ligand and the receptor's active amino acid residues suggested that the compound deserved additional investigation for its potential use in treating COVID-19. The study reported a synthesis process for this compound 31(a-b) (Fig. 21). The procedure involved combining the quinoxaline derivative in an ethanolic solution with diethyl 2-oxo malonate. This mixture was stirred continuously for 2 hours while heated under reflux at 80 °C. The synthesis was completed with a reaction yield of 65%.^31^

**Fig. 22:**
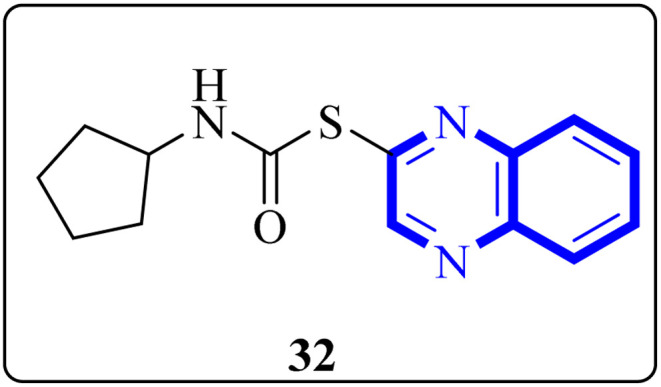
Structure of quinoxaline derivatives compound 32.^32^

Mahgoub, R.E. and collaborators conducted a virtual screening focused on structure, wherein they processed approximately 3.8 million compounds from four distinct chemical libraries to pinpoint inhibitors targeting the active site of the SARS-CoV-2 Mpro enzyme using simple enzyme assays and kinetics studies (a fluorescence resonance energy transfer (FRET)-based assay). Among the substances evaluated, three promising inhibitors were identified, with the quinoxaline derivative known as compound 32 (Fig. 22), showing particularly favorable pharmacokinetic properties, indicative of its potential as a lead compound with IC_50_ value about 301.0 μM against SARS-CoV-2 Mpro. The research determined that compound 32 can fit snugly within the Mpro's active site despite its relatively diminutive structure, engaging crucial subsites effectively. Given its inhibitory potency and characteristics akin to a lead compound, compound 32 represents a viable candidate for further research and development into antiviral medications that could benefit clinical settings.^32^

**Fig. 23:**
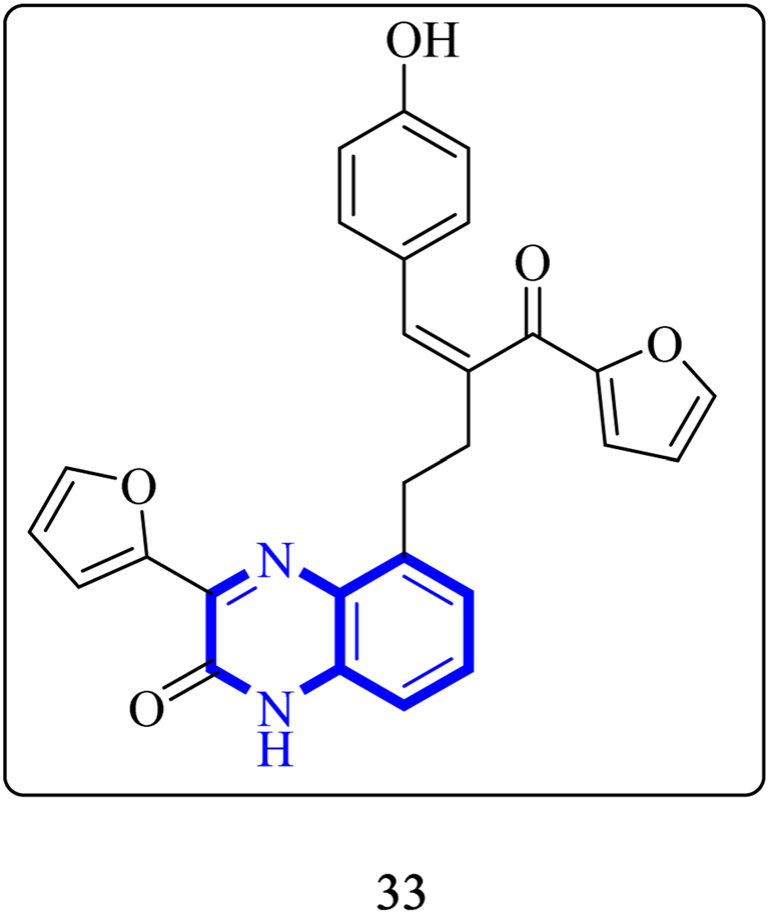
Structure of (*Z*)-5-(3-(furan-2-carbonyl)-4-(4-hydroxyphenyl) but-3-en-1-yl)-3-(furan-2-yl)quinoxalin-2(1*H*)-one as potential SARS-CoV-2.^33^

Through molecular docking studies targeting the Mpro enzyme, a panel of 13 molecules was identified, each adhering to Lipinski's rule of five and demonstrating favorable pharmacokinetic predictions by the QikProp module of Maestro. Among these, the compound designated as (*Z*)-5-(3-(furan-2-carbonyl)-4-(4-hydroxyphenyl)but-3-en-1-yl)-3-(furan-2-yl)quinoxalin-2(1*H*)-one, referred to as compound 33 (Fig. 23), was highlighted. These molecules were subjected to further molecular docking analysis against the SARS-CoV-2 Mpro, reinforcing their promise as possible inhibitors of the SARS-CoV-2 main protease. This *in silico* fragment-based drug design approach highlights these fragments as potential leads for continued research in the drug discovery process aimed at combating COVID-19.^33^

**Fig. 24:**
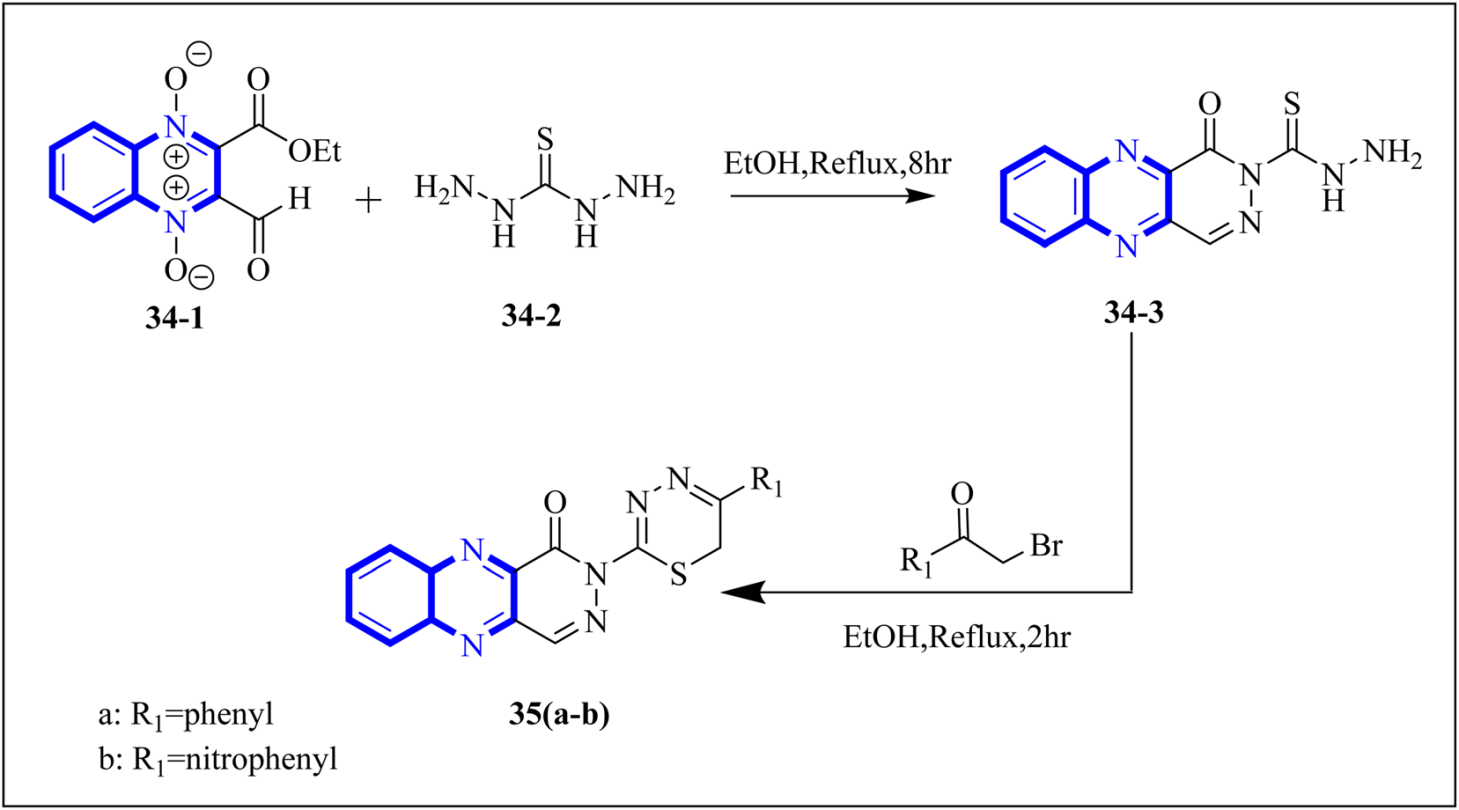
Synthetic route 2-(5-phenyl-6*H*-1,3,4-thiadiazin-2-yl)pyridazino[4,5-*b*]quinoxalin-1(2*H*)-ones, 35-a, 35-b.^7^

P. Moghimi and colleagues focused on pyridazino[4,5-*b*]quinoxaline-1(2*H*)-one structure, specifically compounds 35(a-b), which were evaluated for their effectiveness against the main protease of COVID-19. Molecular docking studies revealed that these compounds, particularly the phenyl and nitrophenyl-substituted variants, displayed strong binding affinities with values of −7.6 kcal mol^−1^ for both. Notably, the spatial arrangement of compounds 35(a-b) within the chymotrypsin-like cysteine protease, 3CL^pro^, was considered advantageous. Additionally, MD simulations aimed at assessing the stability of the protein-ligate complex indicated the nitrophenyl compound formed a stronger association than its phenyl counterpart, suggesting its superior inhibitory action based on the *in silico* analysis. The synthetic pathway for these pyridazino[4,5-*b*]quinoxaline-1(2*H*)-one derivative began with a reaction between compound 37-1 and thiocarbohydrazide, and 34-2 executed in the presence of ethanol under reflux. This process yielded 1-oxopyridazino[4,5-*b*]quinoxaline-2(1*H*)carbothiohydrazide, designated 37-3. Subsequently, compounds 35(a-b) were synthesized using a direct and efficient method) Fig. 24), which involved the reaction of 2-(ethoxycarbonyl)-3-formyl quinoxaline 1,4-dioxide with thiocarbohydrazide under reflux conditions.^7^

**Fig. 25:**
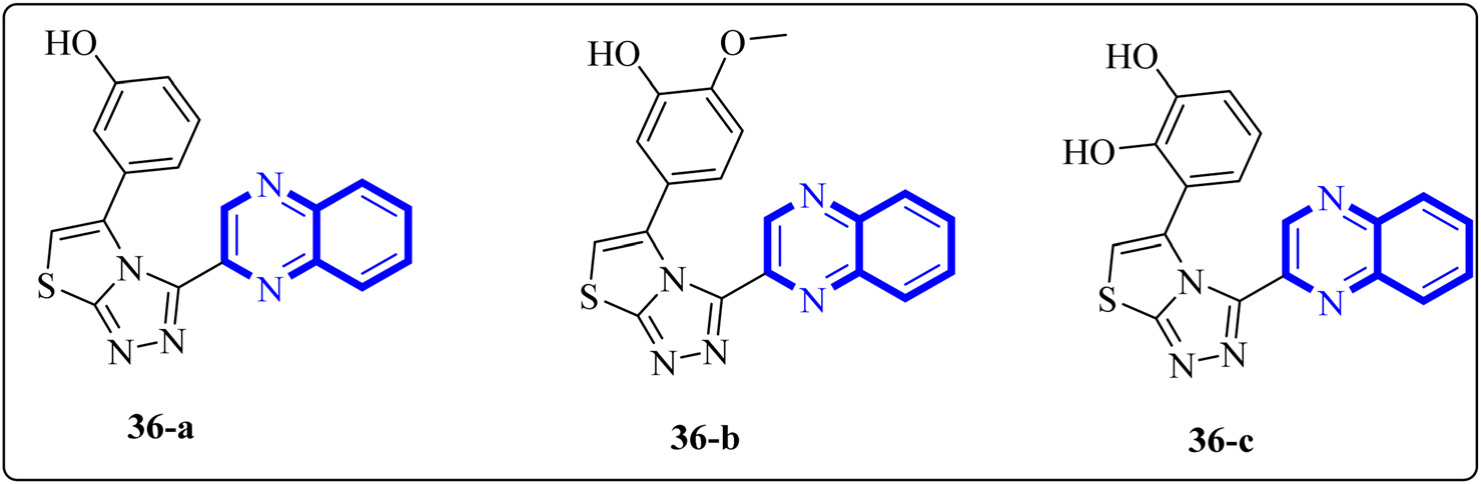
The structure of quinoxaline thiazolo[2,3-*c*] derivatives.^34^

In a study by Ghufran M. *et al.*, potential inhibitors for the SARS-CoV-2 main protease (Mpro) were sought using structure-based virtual screening, molecular docking (*via* molecular operating environment (MOE)), and assessment of drug-like properties. The team employed three distinct databases—ChemBridge, ZINC, and a proprietary in-house collection to identify viable drug candidates that could effectively interact with the catalytic site of Mpro. Among the screened compounds, a quinoxaline derivative labeled as 36-a from the in-house database emerged as the most promising, exhibiting a high docking score of −38.7102 and a root-mean-square deviation (RMSD) value around 2.4 Å across a 150 nanosecond (ns) simulation. The docking conformation suggested that this quinoxaline derivative is expected to establish hydrophilic and hydrophobic interactions with Mpro's active site residues. Additionally, amino acid residues within the enzyme's active site appeared capable of forming hydrogen bonds with the 1,4-dihydroquinoxaline portion of the compound. These findings support the potential application of these compounds (Fig. 25) in the treatment of the SARS-CoV-2 infection.^34^

### 2.4 Miscellaneous

**Fig. 26:**
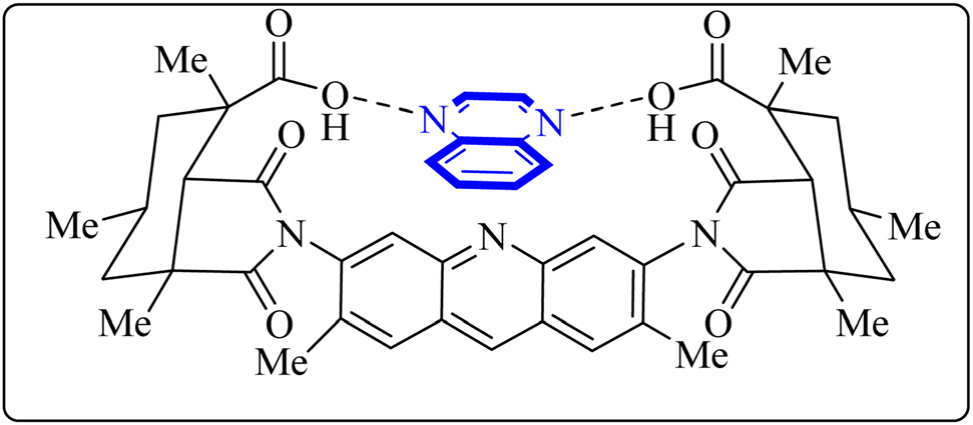
Structure of complex Rebek's acridine diacid with quinoxaline.^37^

Utilizing the EUDOC computational platform, an extensive screen of 23, 426 chemical structures was performed at a granular level of detail—1.0 Å for translational adjustments and 10° for rotational changes—to identify inhibitors of the chymotrypsin-like cysteine protease linked to the severe acute respiratory syndrome-associated coronavirus. Within this research, the complex formation of Rebek's acridine diacid with quinoxaline^35,36^ (Fig. 26) (bearing the Cambridge Structural Database code: YAWJIP) was re-enacted through EUDOC, capitalizing on the non-covalent interaction parameters furnished by the second-generation AMBER force field. Historically, NMR spectroscopy of the YAWJIP complex had delineated a face-to-face π stacking between the guest quinoxaline molecule and the acridine moiety of Rebek's acridine diacid. However, the EUDOC program's three-dimensional reconstructions refined the representations of quinoxaline and Rebek's acridine diacid to establish a complex strikingly similar to the known crystal structure (Form A), presenting a minimal weighted root mean square deviation (mwRMSD) of 0.7 Å. Contrary to earlier suppositions, it did not depict the near face-to-face π-stacking scenario proposed. In conclusion, the employment of the EUDOC program demonstrated its utility in accurately predicting the three-dimensional modeling of host–guest complexes, offering significant contributions to the formulation of novel molecular designs grounded in supramolecular chemistry.^37^

Carta, A. *et al.* studied new classes of linear *N*-tricyclic compounds, pyrido[2,3-*g*]quinoxalines in cell-based assays for cytotoxicity and antiviral activity against the respiratory syncytial virus (RSV). They showed moderate, although particular activity (pyridoquinoxalines 37-b and 37-c against RSV (EC_50_ range = 12–18 μM)). Pyridoquinoxalines (37-b and 37-c derivatives) were developed through the introduction of different electron-withdrawing substituents for the phenyl moiety. SAR studies of pyridoquinoxalines suggested that a phenyl or benzyl side chain for this nucleus is generally more favorable than an aliphatic substituent. Among the studied compounds, 2-oxo-substituted derivatives exhibited higher activity than unsubstituted replications. In addition, pyridoquinoxalines could be designed by introducing different electron-withdrawing substituents on the phenyl moiety.

**Fig. 27:**
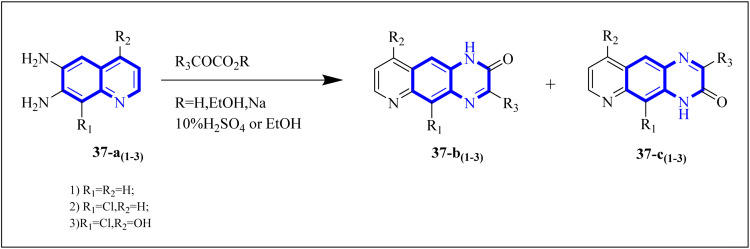
Synthesis pathway of Pyrido quinoxalines derivatives.^38^

The synthetic route of 37-b and 37-c series started following: diamines 37-a series reacted with a-keto carboxylic derivatives in refluxing ethanol for 3–15 h or in 10% aqueous solution of sulfuric acid at 45–50 centigrade for 2 h, combinations of two 2/3-oxo-isomers were obtained in 10–80% yield that named 37-b and 37-c series (Fig. 27) which were then split and purified by chromatography.^38^

### 2.5 The structure–activity relationship (SAR)

#### 2.5.1 The SAR for anti-influenza activity

**Fig. 28:**
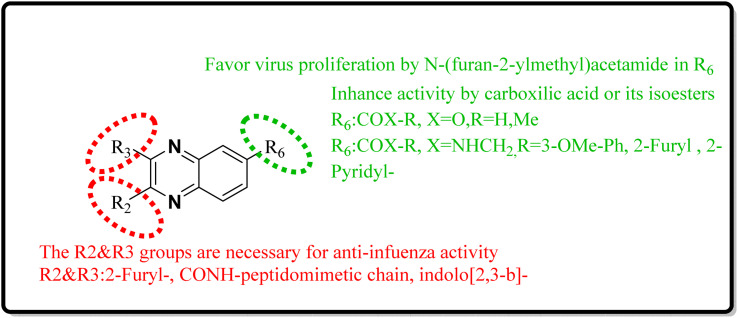
SAR of novel quinoxaline derivatives with anti-influenza activity.

SAR of quinoxaline derivatives in targeting the NS1A protein for anti-influenza therapies was considerably influenced by changes at positions 2 and 3, particularly by bis-2-furyl substitutions (Fig. 28). The study also discovered that introducing various substitutions or heterocycles at position 6 through an amide linker considerably impacted biological activity. Additionally, substituting indole on positions 2 and 3 can enhance the action against influenza.^24,25^

#### 2.5.2 The structure–activity relationship for anti-SARS-CoV activity

**Fig. 29:**
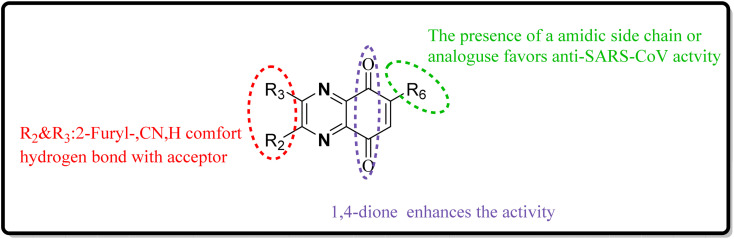
SAR of novel quinoxaline derivatives with anti-SARS corona activity.

**Fig. 30:**
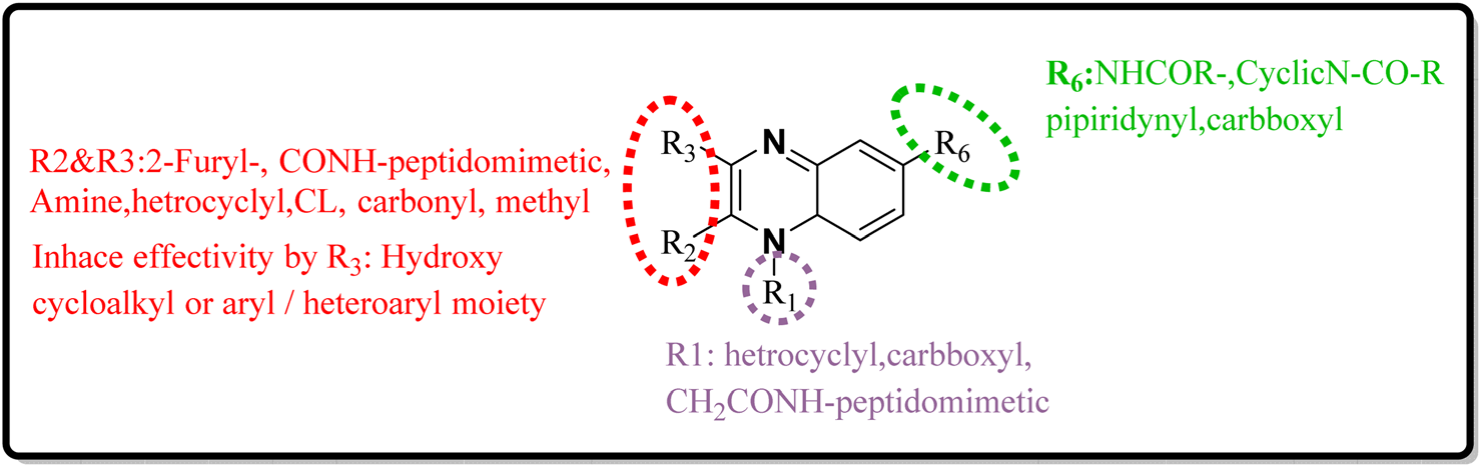
SAR of novel quinoxaline derivatives with anti-SARS-CoV-2 activity.

SAR investigations have identified that an amidic side chain or the presence of isoesters commonly enhances hydrogen bonding interactions with the protein targets of SARS-CoV (Fig. 29). Within this structural context, the quinoxaline core forms a hydrogen bond *via* its nitrogen–hydrogen (–N.H.) group with the arginine residues (specifically Arg55 and Arg164) on the SARS-CoV protein. It was indicated that hydrophobic part of compound 12-c could played key role in PPIase activity of CypA inhibition for example furan ring interacted with the benzene ring of Phe113 *via* π–π stacking. Also the quinoxaline ring by –NH–π hydrogen bond with the –N–H group of Arg55 as it mentioned which was a key determinant against the PPIase activity could be a promising scaffold. Compounds such as 12-c, 14, and 15 have been shown to mimic effectively the binding pattern observed in the ribonucleotide-binding site of the SARS-CoV, suggesting their potential to hinder the viral activity (Fig. 30).^2,16^

#### 2.5.3 The structure–activity relationship for anti-SARS-CoV-2

Analysis conducted on imidazo[1,5-*a*]quinoxalines for SAR purposes indicated an increment in antagonistic activity at position R6, specifically when the side chain had an alkyl length of 4 to 5 carbon atoms, including butyl, pentyl, and isopentyl groups.^25^ Within compound 25(a–c), the SAR study revealed optimal activity when hydroxycycloalkyl, aryl, and heteroaryl groups occupied the R_3_ position alongside a propargyl group. Incorporating an enlarged heterocyclic segment into the quinoxaline core reduced the molecule's flexibility and peptide-like characteristics, enhancing structural stability.^3^ Compounds 27-a and 27-b showed lone pair–p interactions facilitating their binding within the pocket. For 27-b, the presence of 1,4-dihydroquinoxaline provided an additional hydrogen bond donor to the backbone carbonyl of the targeted protein.^17^ The side chain's protonated nitrogen in compound 29 was found to engage in π–cation interactions with the target, augmenting binding space stability. The SAR findings suggested beneficial effects from hydrophobic structures on the tricyclic core, including the substitution of a benzene ring for a pyridine ring, which was well accommodated as evidenced by the binding interactions of compound 29's protonated nitrogen side chain within the pocket.^18^ Molecular docking results for NMPOQA have posited that this ligand, containing N and O atoms with available lone pairs, demonstrated affinity profiles superior to that of the drug Remdesivir, potentially due to its ability to engage in π⋯π stacking as part of hydrophobic interaction. NMPOQA's comparatively lower binding energy suggests it could be a formidable contestant in the quest for COVID-19 therapeutics.^30^ Evaluation of compound 32 revealed that its diminutive size did not preclude it from filling essential subsites of the active site, showcasing a variety of inhibitory and lead-like qualities conducive to the development of clinically significant antiviral agents.^32^ An *in silico* comparative analysis pointed out a more potent inhibitory effect exerted by the nitrophenyl complex over its phenyl counterpart.^3^ The docking behavior of ligand 36-a emphasized the potential for forming dual-natured (hydrophilic and hydrophobic) interactions with the essential amino acid residues in the Mpro protein's activity domain. Furthermore, the proclivity of amino-acid residues to establish hydrogen bonds with the 1,4-dihydroquinoxaline segment of the molecule underscored the therapeutic promise of these compounds, against SARS-CoV-2 ^34^ (Fig. 25).^34^ The detailed structural characteristics and specifications of all compounds investigated in this study are comprehensively listed in Table 1.

## 3. Conclusion

Quinoxaline describes a significant category of nitrogen-containing heterocycles with a wide range of biological activities, especially respiratory antiviral activities. Two-ring hetero-aromatic, quinoxaline derivatives are essential precursors for synthesizing various physiologically significant and pharmacologically utilized molecules. Quinoxaline scafolsshowed potential influenza inhibitory, anti-SARS coronavirus inhibitory, anti-SARS-CO2 coronavirus, and other related anti-virus activities. This present article provides the researchers with synthesized methods on quinoxaline templates reported as respiratory antiviral compounds and a thorough understanding of their effects in helping against significant and epidemic viral diseases such as the COVID-19 pandemic. As we detected, a suitable substitution pattern suggests that the positions of 2, 3 and 6 are significant points of biological relevance for improved activities across antiviral diseases that are studied in this article. Quinoxaline derivatives can be a valuable framework for developing novel pharmacological entities for the treatment of these antiviral diseases in therapeutic medicine. Further, *in vitro* and *in vivo* experimental activities are required to convert these potential inhibitors into clinical drugs. This review could impact the development of therapeutic agents for future antiviral outbreaks such as COVID-19.

**Table 1:** The action mechanism of quinoxaline derivatives in respiratory antiviral activities

Mechanism	No.	Quinoxaline derivatives	Targets (IC_50_ (μM))	Targets or assay	Ref.
Anti-influenza	1	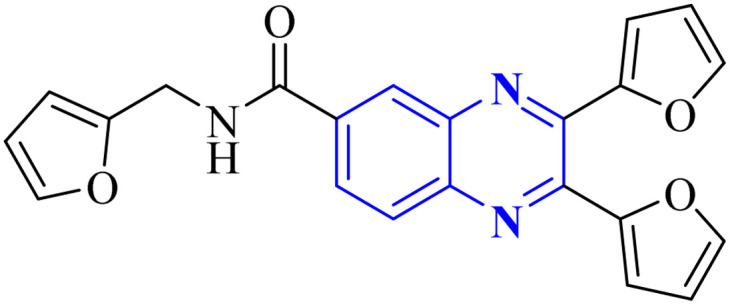	3.5	_	14
	2	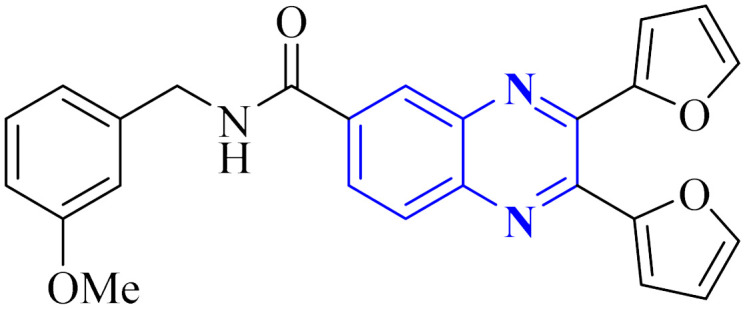	6.2	_	14
	3	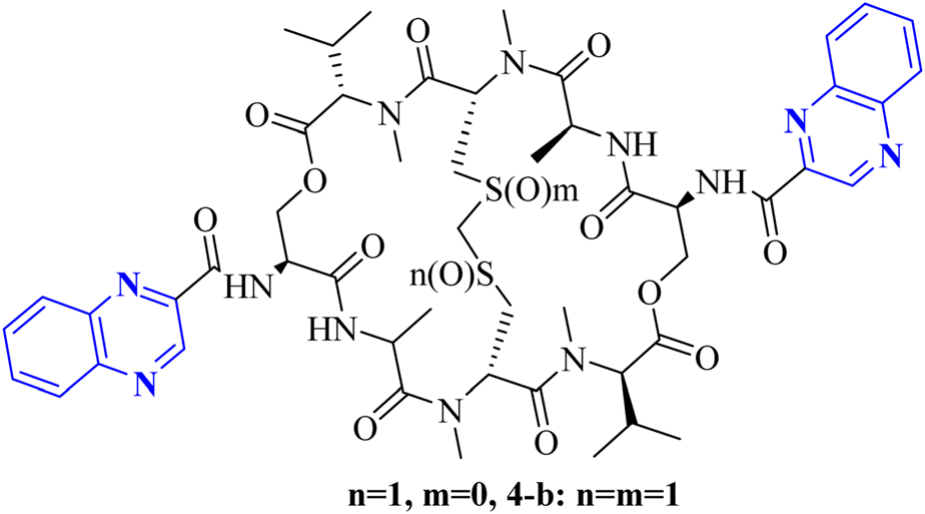	MIC range 0.5–8 mg mL^−1^	VRE (vancomycin-resistant enterococci)	1 and 23
	4	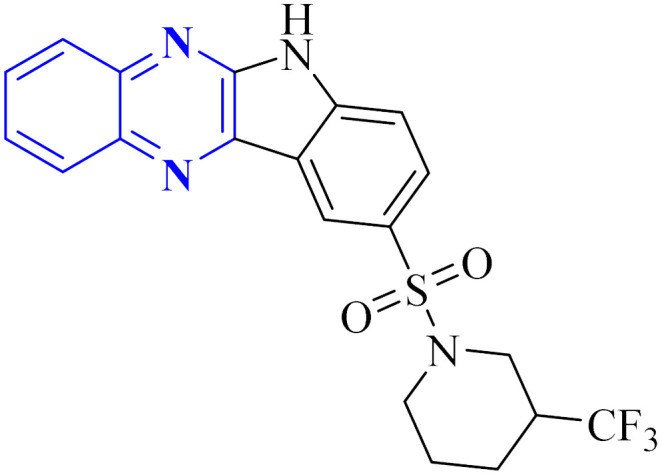	37.04	—	24
SARS corona virus inhibitors	5	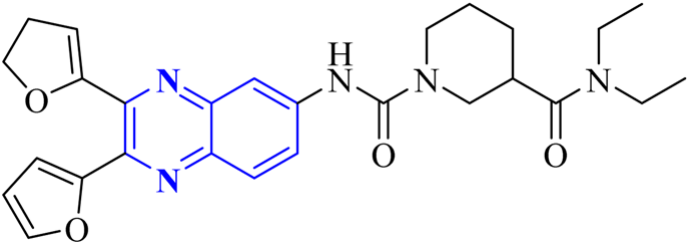	0.41	_	2
	6	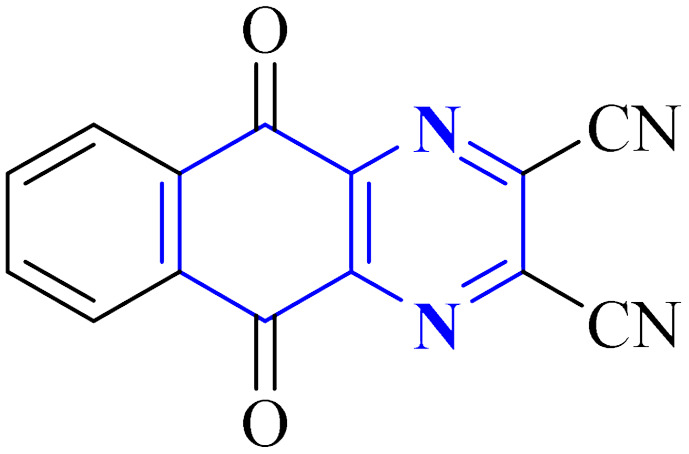	>100	USP8 activity assay	26
SARS corona virus inhibitors	7	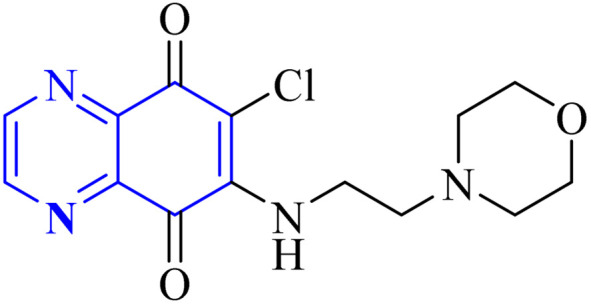	*In silico*	CoV N.P. inhibitory	16
Anti-SARS-CO-2 corona virus	8	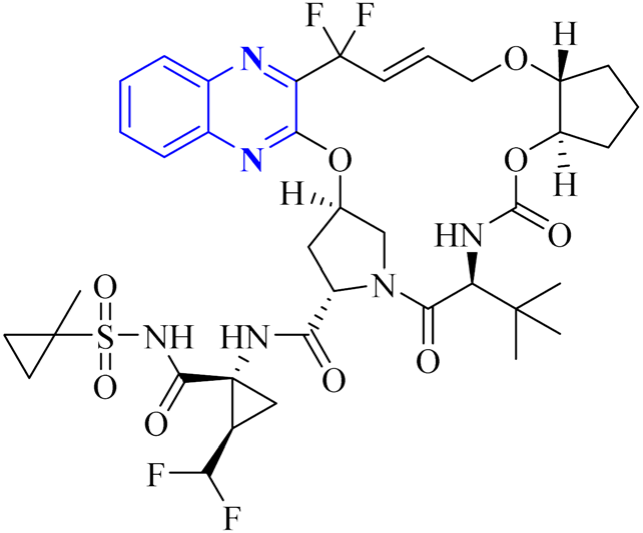	_	_	4
	9	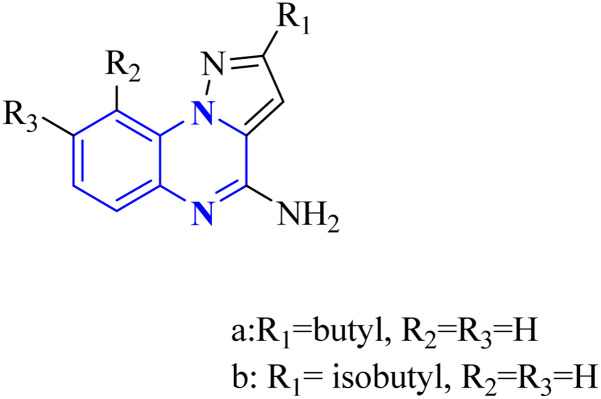	23-a = 8.2	TLR7 antagonist	5
			23-b = 10.0		
	10	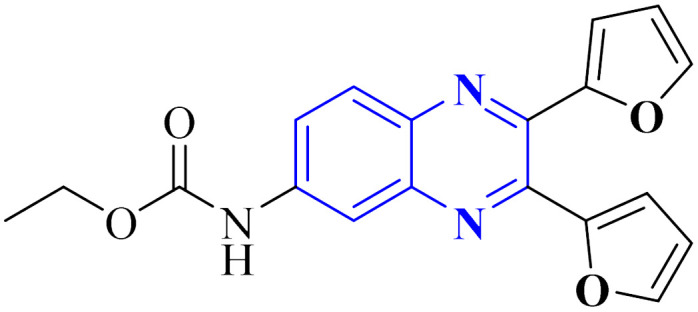	*In silico*	CoV N.P. inhibitory	29
	11	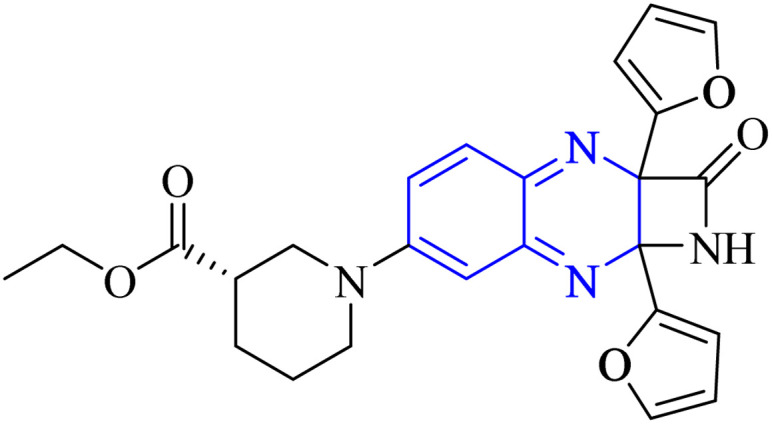	*In silico*	—	29
	12	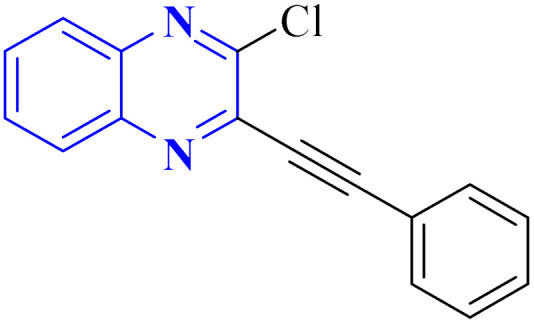	*In silico*	NTD of N-protein of SARS-CoV-2	3
Anti-SARS-CO-2 corona virus	13	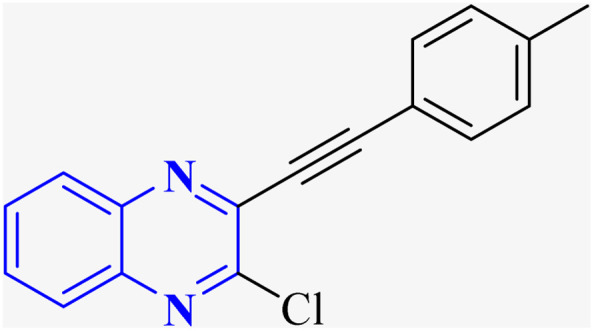			3
	14	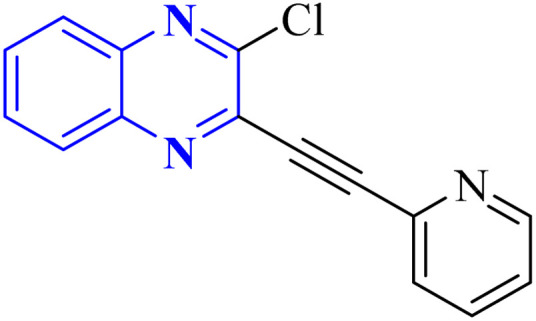	*In silico*	NTD of N-protein of SARS-CoV-2	3
	15	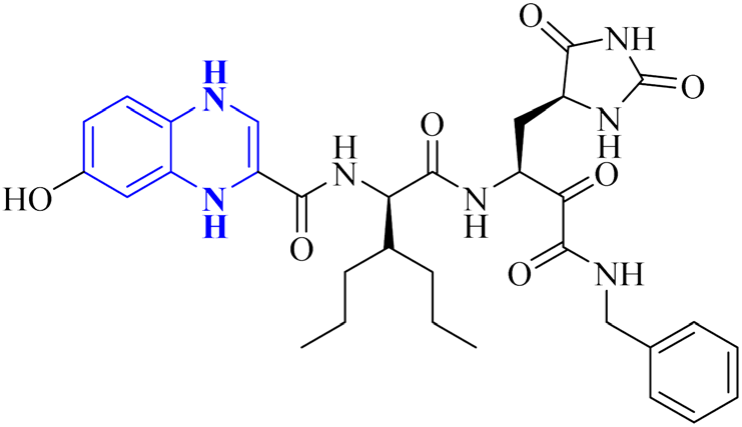	_	_	17
	16	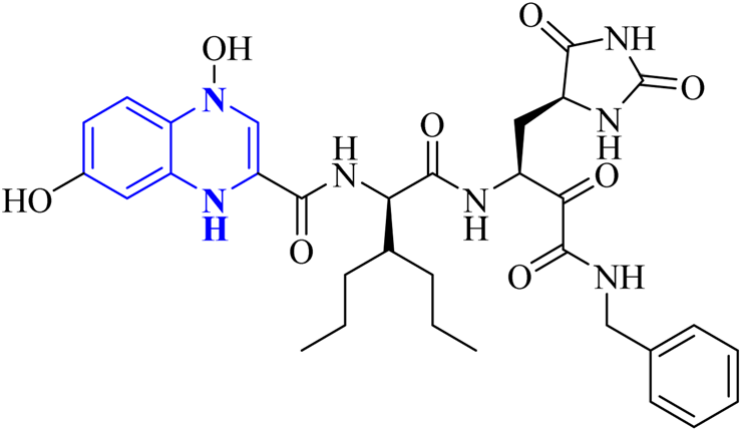	_	_	17
	17	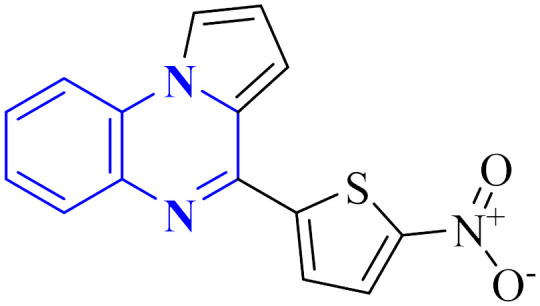	_	*In silico*	6
Anti-SARS-CO-2 corona virus	18	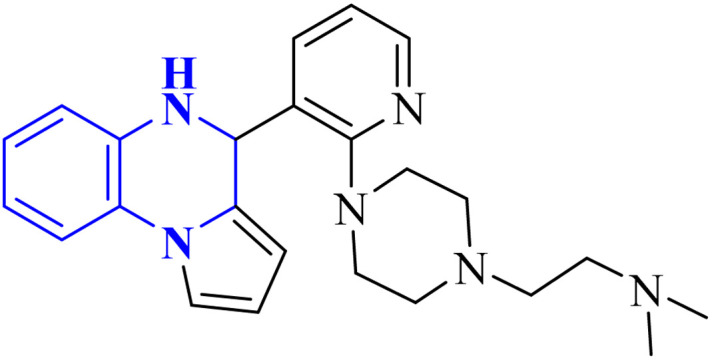	9.3	*In silico*	18
	19	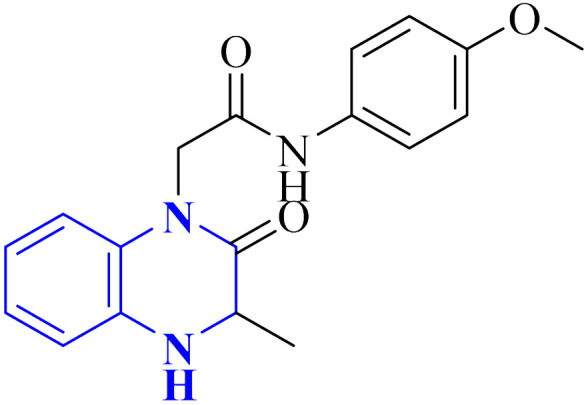	—	*In silico*	30
	20	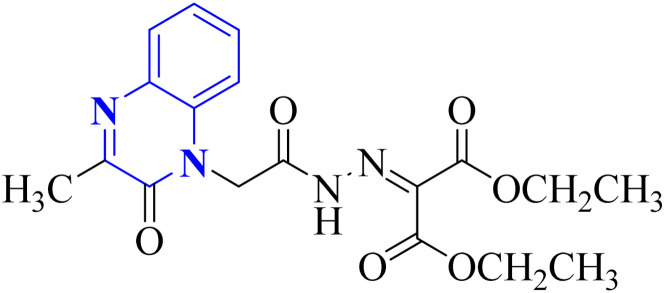	_	*In silico*	31
	21	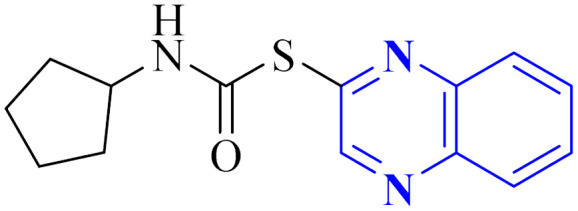	_	*In silico*	32
	22	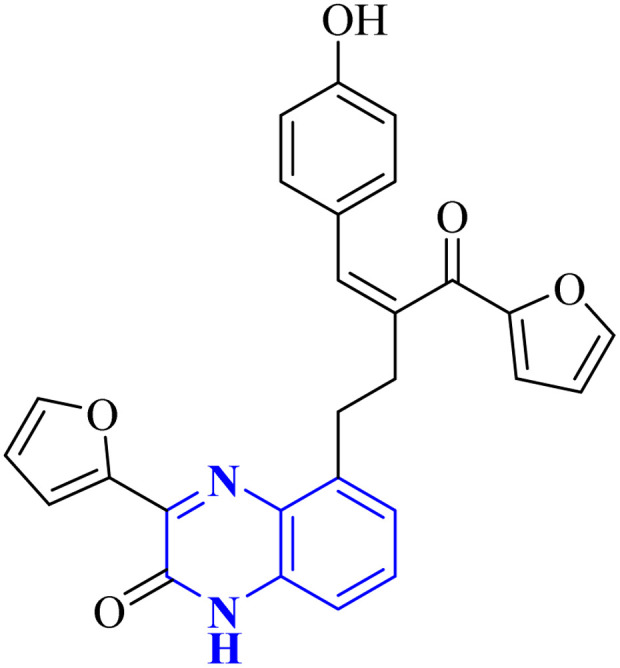	_	*In silico*	33
	23	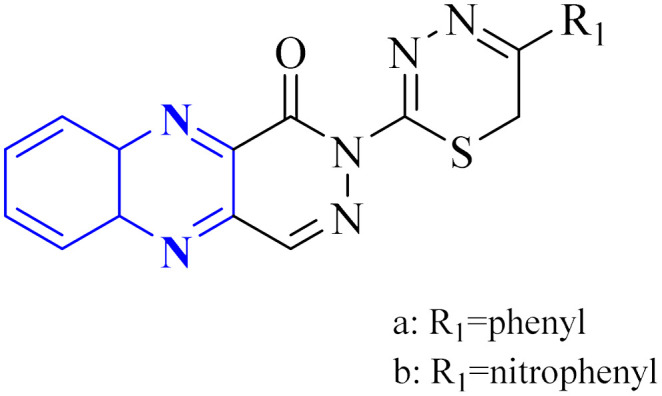	_	*In silico*	7
Anti-SARS-CO-2 corona virus	24	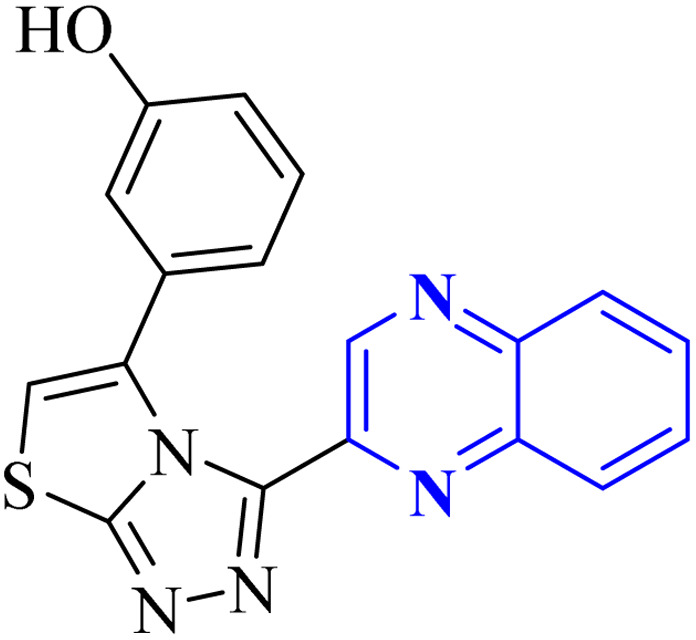	—	*In silico*	34
Miscellaneous	25	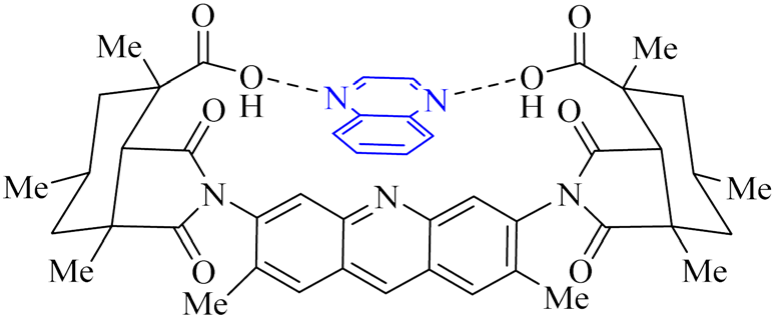	Severe acute respiratory syndrome associated coronavirus	*In silico*	37
	26	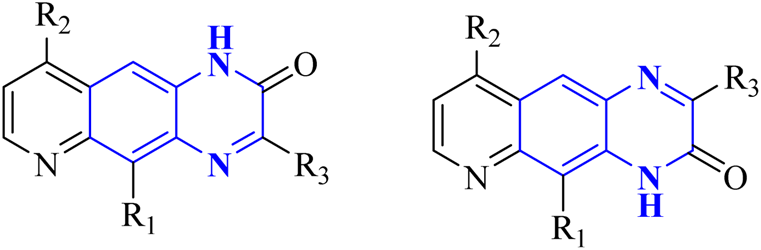	Respiratory syncytial virus (RSV)	_	38
			I_C50_ range = 12–18		

## Conflicts of interest

There are no conflicts to declare.
